# Supplementary material for: High extracellular lactate causes reductive carboxylation in breast tissue cell lines grown under normoxic conditions
Source: PLoS One. 2019 Jun 10;14(6):e0213419. doi: 10.1371/journal.pone.0213419 (PMC6557470; doi:10.1371/journal.pone.0213419)
Supplement: S1 Appendix — (PDF) [file pone.0213419.s001.pdf]

# Supporting information

**Title:** High extracellular lactate increases reductive carboxylation in breast tissue cell lines grown under normoxic conditions
**Authors:** Brodsky, Arthur Nathan, Odenwelder, Daniel C., and Harcum, Sarah W.

**Material Included**
Measured extracellular metabolite concentrations for each cell line (Tables A-C)
Metabolic reactions (Table D)
Schematic of metabolism of labeled by lactate through the TCA cycle (Fig A)
Measured intracellular MIDs for the three tracers for the three cell lines (Tables E-G)
Metabolite isotope labeling distributions for the three cell lines (Figs B and C)
Metabolic flux maps for the three cell lines comparing the two conditions (Figs D-F)
Metabolic flux analysis results for the three cell lines (Tables H-N)
Measured and simulated MIDs for intracellular metabolites for the three cell lines (Figs G-R)

**Table A.** Measured extracellular metabolite concentrations for the **MCF 10A** breast cell line at 24-h and 48-h after the media exchange. Averages are biological triplicates for the amino acid concentrations and biological 6-replicates for glucose and lactate. Standard deviations are provided for these averages.

| **Cell Lines** | **Average Concentration (mM)** | | | | | | | |
|---|---|---|---|---|---|---|---|---|
| | **MCF 10A** | | | | | | | |
| **Condition** | **Control** | | | | **High-Lactate** | | | |
| **Metabolite** | **24-h** | **SD** | **48-h** | **SD** | **24-h** | **SD** | **48-h** | **SD** |
| Glucose (N=6) | 4.3 | 0.248 | 3.31 | 0.345 | 4.42 | 0.173 | 3.63 | 0.332 |
| Lactate (N=6) | 1.12 | 0.116 | 2.81 | 0.139 | 10.8 | 0.217 | 11.9 | 0.271 |
| Glutamine (N=3) | 2.72 | 0.026 | 2.39 | 0.07 | 2.73 | 0.034 | 2.37 | 0.06 |
| **Amino Acids (N=3)** | | | | | | | | |
| Alanine | 0.03 | 0.001 | 0.08 | 0.002 | 0.03 | 0.001 | 0.08 | 0.003 |
| Aspartate | 0.01 | 0.001 | 0.04 | 0.003 | 0.01 | 0.001 | 0.03 | 0.002 |
| Glutamate | 0.03 | 0.003 | 0.09 | 0.005 | 0.03 | 0.003 | 0.07 | 0.003 |
| Glycine | 0.38 | 0.006 | 0.41 | 0.007 | 0.38 | 0.02 | 0.4 | 0.013 |
| Isoleucine | 0.7 | 0.012 | 0.66 | 0.009 | 0.69 | 0.008 | 0.66 | 0.006 |
| Leucine | 0.7 | 0.012 | 0.67 | 0.007 | 0.7 | 0.009 | 0.67 | 0.015 |
| Methionine | 0.18 | 0.004 | 0.17 | 0.006 | 0.17 | 0.004 | 0.17 | 0.003 |
| Phenylalanine | 0.35 | 0.009 | 0.33 | 0.01 | 0.35 | 0.005 | 0.33 | 0.008 |
| Proline | 0.01 | 0.001 | 0.01 | 0.001 | 0 | 0.001 | 0.01 | 0.001 |
| Serine | 0.31 | 0.006 | 0.23 | 0.008 | 0.3 | 0.013 | 0.21 | 0.006 |
| Threonine | 0.71 | 0.004 | 0.71 | 0.006 | 0.71 | 0.004 | 0.71 | 0.005 |
| Tyrosine | 0.35 | 0.009 | 0.33 | 0.006 | 0.35 | 0.006 | 0.33 | 0.012 |
| Valine | 0.71 | 0.007 | 0.68 | 0.009 | 0.7 | 0.005 | 0.68 | 0.007 |

**Table B.** Measured extracellular metabolite concentrations for the **MCF7** breast cell line at 24-h and 48-h after the media exchange. Averages are biological triplicates for the amino acid concentrations and biological 6-replicates for glucose and lactate. Standard deviations are provided for these averages.

| Cell Lines | Average Concentration (mM) | | | | | | | |
|---|---|---|---|---|---|---|---|---|
| | MCF 7 | | | | | | | |
| **Condition** | **Control** | | | | **High-Lactate** | | | |
| **Metabolite** | **24-h** | **SD** | **48-h** | **SD** | **24-h** | **SD** | **48-h** | **SD** |
| Glucose (N=6) | 4.32 | 0.206 | 3.32 | 0.231 | 4.5 | 0.195 | 3.78 | 0.250 |
| Lactate (N=6) | 1.03 | 0.100 | 2.55 | 0.131 | 20.5 | 0.108 | 21.1 | 0.193 |
| Glutamine (N=3) | 2.72 | 0.046 | 2.35 | 0.060 | 2.71 | 0.034 | 2.31 | 0.051 |
| **Amino Acids (N=3)** | | | | | | | | |
| Alanine | 0.04 | 0.002 | 0.1 | 0.004 | 0.03 | 0.004 | 0.09 | 0.004 |
| Aspartate | 0.02 | 0.002 | 0.05 | 0.005 | 0.01 | 0.002 | 0.04 | 0.003 |
| Glutamate | 0.04 | 0.003 | 0.09 | 0.005 | 0.04 | 0.003 | 0.09 | 0.003 |
| Glycine | 0.39 | 0.016 | 0.44 | 0.013 | 0.39 | 0.009 | 0.43 | 0.017 |
| Isoleucine | 0.69 | 0.010 | 0.65 | 0.003 | 0.69 | 0.008 | 0.65 | 0.008 |
| Leucine | 0.7 | 0.005 | 0.66 | 0.018 | 0.7 | 0.009 | 0.66 | 0.009 |
| Methionine | 0.17 | 0.004 | 0.16 | 0.007 | 0.17 | 0.003 | 0.16 | 0.005 |
| Phenylalanine | 0.35 | 0.006 | 0.33 | 0.007 | 0.35 | 0.004 | 0.33 | 0.009 |
| Proline | 0.01 | 0.001 | 0.02 | 0.002 | 0.01 | 0.001 | 0.02 | 0.001 |
| Serine | 0.3 | 0.015 | 0.2 | 0.012 | 0.29 | 0.018 | 0.19 | 0.013 |
| Threonine | 0.72 | 0.001 | 0.71 | 0.004 | 0.72 | 0.002 | 0.71 | 0.004 |
| Tyrosine | 0.35 | 0.005 | 0.33 | 0.007 | 0.35 | 0.005 | 0.33 | 0.009 |
| Valine | 0.69 | 0.006 | 0.65 | 0.009 | 0.69 | 0.008 | 0.64 | 0.018 |

**Table C.** Measured extracellular metabolite concentrations for the **MDA-MB-231** breast cell line at 24-h and 48-h after the media exchange. Averages are biological triplicates for the amino acid concentrations and biological 6-replicates for glucose and lactate. Standard deviations are provided for these averages.

| Cell Lines | Average Concentration (mM) | | | | | | | |
|---|---|---|---|---|---|---|---|---|
| | MDA-MB-231 | | | | | | | |
| **Condition** | **Control** | | | | **High-Lactate** | | | |
| **Metabolite** | **24-h** | **SD** | **48-h** | **SD** | **24-h** | **SD** | **48-h** | **SD** |
| Glucose (N=6) | 4.05 | 0.233 | 2.38 | 0.238 | 4.2 | 0.124 | 2.9 | 0.247 |
| Lactate (N=6) | 1.8 | 0.206 | 4.8 | 0.242 | 21 | 0.202 | 22.7 | 0.323 |
| Glutamine (N=3) | 2.75 | 0.047 | 2.42 | 0.079 | 2.73 | 0.045 | 2.34 | 0.061 |
| **Amino Acids (N=3)** | | | | | | | | |
| Alanine | 0.03 | 0.002 | 0.08 | 0.004 | 0.03 | 0.002 | 0.07 | 0.003 |
| Aspartate | 0.01 | 0.001 | 0.03 | 0.003 | 0.01 | 0.001 | 0.03 | 0.003 |
| Glutamate | 0.03 | 0.004 | 0.07 | 0.003 | 0.03 | 0.002 | 0.08 | 0.003 |
| Glycine | 0.38 | 0.008 | 0.42 | 0.011 | 0.38 | 0.010 | 0.42 | 0.013 |
| Isoleucine | 0.71 | 0.009 | 0.68 | 0.007 | 0.7 | 0.007 | 0.65 | 0.009 |
| Leucine | 0.71 | 0.009 | 0.68 | 0.013 | 0.69 | 0.007 | 0.65 | 0.007 |
| Methionine | 0.18 | 0.001 | 0.17 | 0.003 | 0.17 | 0.004 | 0.16 | 0.006 |
| Phenylalanine | 0.35 | 0.002 | 0.34 | 0.006 | 0.35 | 0.002 | 0.33 | 0.014 |
| Proline | 0 | 0.000 | 0.01 | 0.001 | 0 | 0.000 | 0.01 | 0.001 |
| Serine | 0.32 | 0.012 | 0.24 | 0.011 | 0.3 | 0.013 | 0.2 | 0.005 |
| Threonine | 0.72 | 0.001 | 0.71 | 0.005 | 0.71 | 0.002 | 0.71 | 0.006 |
| Tyrosine | 0.35 | 0.010 | 0.33 | 0.007 | 0.35 | 0.004 | 0.33 | 0.011 |
| Valine | 0.70 | 0.009 | 0.67 | 0.006 | 0.69 | 0.002 | 0.65 | 0.007 |

**Table D.** The metabolic reactions used in the $^{13}$C-MFA simulations for the three breast cell lines. Suffixes indicate compartmental location of the metabolite: .ext, extracellular; .c, cytosolic; .m, mitochondrial; .tr, tracer; .snk, dilution sink.

| | |
|---|---|
| v1 | Gluc.ext (abcdef) -> G6P.c (abcdef) |
| v2 | G6P.c (abcdef) <=> F6P.c (net) (abcdef) v3F6P.c (abcdef) -> FBP.c (abcdef) |
| v4 | FBP.c (abcdef) <=>DHAP.c (cba) + GAP.c (def) |
| v5 | DHAP.c (abc) <=> GAP.c (abc) |
| v6 | GAP.c (abc) <=> 3PG.c (abc) |
| v7 | 3PG.c (abc)<=> Pyr.c (abc) |
| v8 | G6P.c (abcdef) -> CO2 (a) + Ru5P.c (bcdef) |
| v9 | Ru5P.c (abcde) <=> R5P.c (abcde) |
| v10 | Ru5P.c (abcde) <=> X5P.c (net) (abcde) |
| v11 | X5P.c (abcde) + R5P.c (fghij) <=> GAP.c (abc) + S7P.c (fghijde) |
| v12 | S7P.c (abcdefg) + GAP.c (hij) <=> E4P.c (abcd) + F6P.c (hijefg) |
| v13 | X5P.c (abcde) + E4P.c (fghi) <=> GAP.c (abc) + F6P (fghide) |
| v14 | Pyr.c (abc) <=> Lact.c (abc) |
| v15 | Pyr.c (abc)-> CO2 (a) + AcCoA.m (bc) |
| v16 | AcCoA.m (ab) + OAA.m (cdef) -> Cit.m (fedbac) |
| v17 | Cit.m (abcdef) <=> AKG.m (abcde) + CO2 (f) |
| v18 | AKG.m (abcde) -> CO2 (a) + Suc.m (bcde) |
| v19 | Suc.m (abcd) <=> Fum.m (abcd) |
| v20 | Fum.m (abcd) <=> Mal.m (abcd) |
| v21 | Mal.m (abcd) <=> OAA.m (abcd) |
| v22 | Gln.c (abcde) -> Glu.c (abcde) |
| v23 | Ser.c (abc) <=> Pyr.c (abc) |

v24 Gln.ext (abcde) -> Gln.c (abcde)

v25 Asp.c (abcd) -> Asp.ext (abcd)

v26 Ser.ext (abc) -> Ser.c (abc)

v27 Ala.c (abc) -> Ala.ext (abc)

v28 Glu.c (abcde) -> Glu.ext (abcde)

v29 Lact.c (abc) -> Lact.ext (abc)

v30 Glu.c (abcde) <=> AKG.m (abcde)

v31 Mal.m (abcd) -> Pyr.c (abc)+ CO2 (d)

v32 Cit.m (abcdef) -> OAA.m (cdef)+ AcCoA.c (ab)

v33 Pyr.c (abc) + CO2 (d) -> OAA.m (abcd)

v34 0.95 AcCoA.c + 0.05 DHAP.c -> Lipid

v35 Pyr.c (abc) + Glu.c (defgh) <=> Ala.c (abc) + AKG.m (defgh)

v36 OAA.m (abcd) + Glu.c (efghi) <=> Asp.c (abcd) + AKG.m (efghi)

v37 0.23 Ala.c + 0.14 Asp.c + 0.12 Gln.c + 0.15 Glu.c + 0.16 Ser.c + 0.11 G6P.c + 0.09 R5P.c -> Biomass

v38 Lact.c (abc) + Lact.tr (def) -> Lact.c (def) + Lact.snk (abc)

**Fig A. Metabolism of labeled lactate through the TCA cycle.** A) M2-labeled TCA intermediates are generated from [U-$^{13}$C] lactate (Lac). B) M1-labeled a-oxoglutarate (AKG) and oxaloacetate (OAA) are generated from unlabeled pyruvate (Pyr) combining with M2-labeled oxaloacetate (OAA). M1 citrate is generated via citrate synthase through the combination of M1-labeled oxaloacetate (OAA) and M0 acetyl-CoA (AcCoA). For clarity, some reaction pathways were condensed.

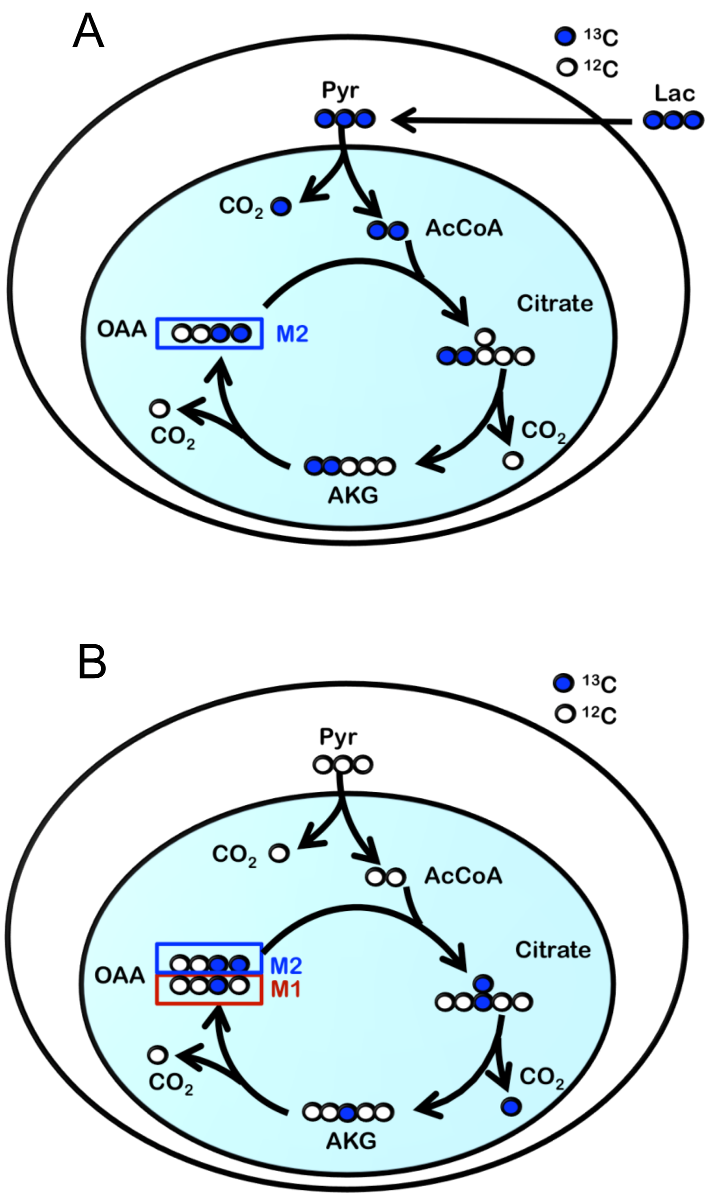

**Table E.** Measured intracellular MIDs from the [1,2-$^{13}$C] glucose tracer for the three breast cell lines for the control and high-lactate conditions with mean and standard errors. MIDs shown below have not been corrected for natural abundance. A standard error of 0.006 represents the lower limit error cutoff reported for most instruments [1, 2], which was applied even when the biological replicates had observed lower standard error.

| Cell Line | MCF 10A | | | | MCF7 | | | | MDA-MB-231 | | | |
|---|---|---|---|---|---|---|---|---|---|---|---|---|
| Condition | Control | | High-Lactate | | Control | | High-Lactate | | Control | | High-Lactate | |
| PEP 453 | MID | SE | MID | SE | MID | SE | MID | SE | MID | SE | MID | SE |
| M0 | 0.3701 | 0.0129 | 0.3501 | 0.0078 | 0.3606 | 0.0119 | 0.3666 | 0.0111 | 0.3459 | 0.0147 | 0.3906 | 0.0060 |
| M1 | 0.1654 | 0.0129 | 0.1515 | 0.0078 | 0.1507 | 0.0119 | 0.1527 | 0.0111 | 0.1527 | 0.0147 | 0.1570 | 0.0060 |
| M2 | 0.3159 | 0.0129 | 0.3333 | 0.0078 | 0.3330 | 0.0119 | 0.3248 | 0.0111 | 0.3339 | 0.0147 | 0.3042 | 0.0060 |
| M3 | 0.0994 | 0.0129 | 0.1045 | 0.0078 | 0.1064 | 0.0119 | 0.1008 | 0.0111 | 0.1097 | 0.0147 | 0.0962 | 0.0060 |
| M4 | 0.0394 | 0.0129 | 0.0502 | 0.0078 | 0.0394 | 0.0119 | 0.0448 | 0.0111 | 0.0477 | 0.0147 | 0.0422 | 0.0060 |
| M5 | 0.0098 | 0.0129 | 0.0104 | 0.0078 | 0.0099 | 0.0119 | 0.0101 | 0.0111 | 0.0101 | 0.0147 | 0.0098 | 0.0060 |
| 3PG 585 | | | | | | | | | | | | |
| M0 | 0.3128 | 0.0083 | 0.3014 | 0.0075 | 0.3119 | 0.0143 | 0.3060 | 0.0142 | 0.3060 | 0.0140 | 0.3018 | 0.0064 |
| M1 | 0.1675 | 0.0083 | 0.1647 | 0.0075 | 0.1650 | 0.0143 | 0.1643 | 0.0142 | 0.1643 | 0.0140 | 0.1653 | 0.0064 |
| M2 | 0.3108 | 0.0083 | 0.3194 | 0.0075 | 0.3119 | 0.0143 | 0.3183 | 0.0142 | 0.3183 | 0.0140 | 0.3202 | 0.0064 |
| M3 | 0.1292 | 0.0083 | 0.1327 | 0.0075 | 0.1308 | 0.0143 | 0.1314 | 0.0142 | 0.1314 | 0.0140 | 0.1322 | 0.0064 |
| M4 | 0.0605 | 0.0083 | 0.0639 | 0.0075 | 0.0603 | 0.0143 | 0.0606 | 0.0142 | 0.0606 | 0.0140 | 0.0610 | 0.0064 |
| M5 | 0.0192 | 0.0083 | 0.0180 | 0.0075 | 0.0201 | 0.0143 | 0.0195 | 0.0142 | 0.0195 | 0.0140 | 0.0196 | 0.0064 |
| DHAP 484 | | | | | | | | | | | | |
| M0 | 0.2323 | 0.0112 | 0.3273 | 0.0100 | 0.3421 | 0.0137 | 0.2781 | 0.0097 | 0.2781 | 0.0139 | 0.3391 | 0.0103 |
| M1 | 0.1413 | 0.0112 | 0.1514 | 0.0100 | 0.1484 | 0.0137 | 0.1379 | 0.0097 | 0.1379 | 0.0139 | 0.1479 | 0.0103 |
| M2 | 0.4147 | 0.0112 | 0.3436 | 0.0100 | 0.3401 | 0.0137 | 0.3893 | 0.0097 | 0.3893 | 0.0139 | 0.3391 | 0.0103 |
| M3 | 0.1404 | 0.0112 | 0.1165 | 0.0100 | 0.1134 | 0.0137 | 0.1290 | 0.0097 | 0.1290 | 0.0139 | 0.1139 | 0.0103 |
| M4 | 0.0617 | 0.0112 | 0.0497 | 0.0100 | 0.0453 | 0.0137 | 0.0556 | 0.0097 | 0.0556 | 0.0139 | 0.0486 | 0.0103 |
| M5 | 0.0096 | 0.0112 | 0.0116 | 0.0100 | 0.0106 | 0.0137 | 0.0101 | 0.0097 | 0.0100 | 0.0139 | 0.0113 | 0.0103 |
| PYR 174 | | | | | | | | | | | | |
| M0 | 0.5318 | 0.0146 | 0.5581 | 0.0071 | 0.5258 | 0.0188 | 0.5350 | 0.0136 | 0.4816 | 0.0132 | 0.5581 | 0.0114 |
| M1 | 0.1062 | 0.0146 | 0.0948 | 0.0071 | 0.0969 | 0.0188 | 0.0975 | 0.0136 | 0.0976 | 0.0132 | 0.0948 | 0.0114 |
| M2 | 0.3125 | 0.0146 | 0.2983 | 0.0071 | 0.3196 | 0.0188 | 0.3170 | 0.0136 | 0.3579 | 0.0132 | 0.2983 | 0.0114 |
| M3 | 0.0320 | 0.0146 | 0.0335 | 0.0071 | 0.0361 | 0.0188 | 0.0345 | 0.0136 | 0.0412 | 0.0132 | 0.0335 | 0.0114 |
| M4 | 0.0134 | 0.0146 | 0.0125 | 0.0071 | 0.0134 | 0.0188 | 0.0129 | 0.0136 | 0.0130 | 0.0132 | 0.0125 | 0.0114 |
| M5 | 0.0041 | 0.0146 | 0.0029 | 0.0071 | 0.0082 | 0.0188 | 0.0029 | 0.0136 | 0.0087 | 0.0132 | 0.0029 | 0.0114 |

| Cell Line | MCF 10A | | | | MCF7 | | | | MDA-MB-231 | | | |
|---|---|---|---|---|---|---|---|---|---|---|---|---|
| Condition | Control | | High-Lactate | | Control | | High-Lactate | | Control | | High-Lactate | |
| PEP 453 | MID | SE | MID | SE | MID | SE | MID | SE | MID | SE | MID | SE |
| ALA 260 | | | | | | | | | | | | |
| M0 | 0.4656 | 0.0148 | 0.4813 | 0.0111 | 0.4661 | 0.0197 | 0.4654 | 0.0101 | 0.4258 | 0.0131 | 0.4912 | 0.0085 |
| M1 | 0.1453 | 0.0148 | 0.1348 | 0.0111 | 0.1322 | 0.0197 | 0.1347 | 0.0101 | 0.1331 | 0.0131 | 0.1339 | 0.0085 |
| M2 | 0.2993 | 0.0148 | 0.2936 | 0.0111 | 0.3125 | 0.0197 | 0.3069 | 0.0101 | 0.3393 | 0.0131 | 0.2854 | 0.0085 |
| M3 | 0.0616 | 0.0148 | 0.0616 | 0.0111 | 0.0637 | 0.0197 | 0.0634 | 0.0101 | 0.0719 | 0.0131 | 0.0600 | 0.0085 |
| M4 | 0.0234 | 0.0148 | 0.0231 | 0.0111 | 0.0196 | 0.0197 | 0.0237 | 0.0101 | 0.0240 | 0.0131 | 0.0236 | 0.0085 |
| M5 | 0.0048 | 0.0148 | 0.0058 | 0.0111 | 0.0059 | 0.0197 | 0.0059 | 0.0101 | 0.0060 | 0.0131 | 0.0059 | 0.0085 |
| LAC 261 | | | | | | | | | | | | |
| M0 | 0.4668 | 0.0124 | 0.4862 | 0.0102 | 0.4611 | 0.0197 | 0.4926 | 0.0112 | 0.4233 | 0.0148 | 0.4971 | 0.0152 |
| M1 | 0.1451 | 0.0124 | 0.1335 | 0.0102 | 0.1332 | 0.0197 | 0.1360 | 0.0112 | 0.1325 | 0.0148 | 0.1345 | 0.0152 |
| M2 | 0.2987 | 0.0124 | 0.2908 | 0.0102 | 0.3176 | 0.0197 | 0.2838 | 0.0112 | 0.3426 | 0.0148 | 0.2807 | 0.0152 |
| M3 | 0.0612 | 0.0124 | 0.0610 | 0.0102 | 0.0615 | 0.0197 | 0.0581 | 0.0112 | 0.0717 | 0.0148 | 0.0585 | 0.0152 |
| M4 | 0.0234 | 0.0124 | 0.0228 | 0.0102 | 0.0205 | 0.0197 | 0.0236 | 0.0112 | 0.0239 | 0.0148 | 0.0233 | 0.0152 |
| M5 | 0.0048 | 0.0124 | 0.0057 | 0.0102 | 0.0061 | 0.0197 | 0.0059 | 0.0112 | 0.0060 | 0.0148 | 0.0058 | 0.0152 |
| GLP 571 | | | | | | | | | | | | |
| M0 | 0.2072 | 0.0067 | 0.2895 | 0.0086 | 0.2973 | 0.0166 | 0.2415 | 0.0100 | 0.2394 | 0.0145 | 0.2976 | 0.0121 |
| M1 | 0.1437 | 0.0067 | 0.1619 | 0.0086 | 0.1589 | 0.0166 | 0.1449 | 0.0100 | 0.1455 | 0.0145 | 0.1601 | 0.0121 |
| M2 | 0.3812 | 0.0067 | 0.3278 | 0.0086 | 0.3226 | 0.0166 | 0.3622 | 0.0100 | 0.3606 | 0.0145 | 0.3229 | 0.0121 |
| M3 | 0.1642 | 0.0067 | 0.1369 | 0.0086 | 0.1365 | 0.0166 | 0.1531 | 0.0100 | 0.1559 | 0.0145 | 0.1361 | 0.0121 |
| M4 | 0.0811 | 0.0067 | 0.0648 | 0.0086 | 0.0682 | 0.0166 | 0.0768 | 0.0100 | 0.0770 | 0.0145 | 0.0644 | 0.0121 |
| M5 | 0.0225 | 0.0067 | 0.0191 | 0.0086 | 0.0166 | 0.0166 | 0.0214 | 0.0100 | 0.0216 | 0.0145 | 0.0189 | 0.0121 |

**Table F.** Measured intracellular MIDs from [U-$^{13}$C] glutamine tracer for the three breast cancer cell lines for the control and high-lactate conditions with mean and standard errors. MIDs shown below have not been corrected for natural abundance. A standard error of 0.006 represents the lower limit error cutoff reported for most instruments [1, 2], which was applied even when the biological replicates had observed lower standard error.

| **Cell Line** | **MCF 10A** | | | | **MCF7** | | | | **MDA-MB-231** | | | |
|---|---|---|---|---|---|---|---|---|---|---|---|---|
| **Condition** | **Control** | | **High-Lactate** | | **Control** | | **High-Lactate** | | **Control** | | **High-Lactate** | |
| | **MID** | **SE** | **MID** | **SE** | **MID** | **SE** | **MID** | **SE** | **MID** | **SE** | **MID** | **SE** |
| **PYR 174** | | | | | | | | | | | | |
| M0 | 0.824 | 0.0088 | 0.8022 | 0.006 | 0.8068 | 0.0158 | 0.8301 | 0.009 | 0.837 | 0.0081 | 0.808 | 0.0103 |
| M1 | 0.1061 | 0.0088 | 0.1124 | 0.006 | 0.1187 | 0.0158 | 0.107 | 0.009 | 0.105 | 0.0081 | 0.111 | 0.0103 |
| M2 | 0.0424 | 0.0088 | 0.0462 | 0.006 | 0.0451 | 0.0158 | 0.04 | 0.009 | 0.042 | 0.0081 | 0.046 | 0.0103 |
| M3 | 0.0242 | 0.0088 | 0.0358 | 0.006 | 0.0261 | 0.0158 | 0.0195 | 0.009 | 0.013 | 0.0081 | 0.033 | 0.0103 |
| M4 | 0.0024 | 0.0088 | 0.0026 | 0.006 | 0.0024 | 0.0158 | 0.0025 | 0.009 | 0.002 | 0.0081 | 0.003 | 0.0103 |
| M5 | 0.0009 | 0.0088 | 0.0009 | 0.006 | 0.0009 | 0.0158 | 0.0009 | 0.009 | 0.001 | 0.0081 | 0.001 | 0.0103 |
| **ALA 260** | | | | | | | | | | | | |
| M0 | 0.7278 | 0.0094 | 0.7047 | 0.0097 | 0.7235 | 0.018 | 0.732 | 0.006 | 0.737 | 0.0083 | 0.713 | 0.007 |
| M1 | 0.1654 | 0.0094 | 0.1693 | 0.0097 | 0.1608 | 0.018 | 0.1637 | 0.006 | 0.165 | 0.0083 | 0.167 | 0.007 |
| M2 | 0.0717 | 0.0094 | 0.0757 | 0.0097 | 0.0769 | 0.018 | 0.0713 | 0.006 | 0.071 | 0.0083 | 0.075 | 0.007 |
| M3 | 0.0276 | 0.0094 | 0.0415 | 0.0097 | 0.031 | 0.018 | 0.025 | 0.006 | 0.02 | 0.0083 | 0.037 | 0.007 |
| M4 | 0.0066 | 0.0094 | 0.0073 | 0.0097 | 0.0069 | 0.018 | 0.0066 | 0.006 | 0.006 | 0.0083 | 0.007 | 0.007 |
| M5 | 0.0009 | 0.0094 | 0.0014 | 0.0097 | 0.0009 | 0.018 | 0.0013 | 0.006 | 0.001 | 0.0083 | 0.001 | 0.007 |
| **LAC 261** | | | | | | | | | | | | |
| M0 | 0.7185 | 0.0072 | 0.6983 | 0.006 | 0.7079 | 0.0165 | 0.7275 | 0.0066 | 0.731 | 0.0081 | 0.708 | 0.0118 |
| M1 | 0.1684 | 0.0072 | 0.173 | 0.006 | 0.1741 | 0.0165 | 0.1679 | 0.0066 | 0.169 | 0.0081 | 0.17 | 0.0118 |
| M2 | 0.0752 | 0.0072 | 0.0774 | 0.006 | 0.0812 | 0.0165 | 0.0727 | 0.0066 | 0.072 | 0.0081 | 0.076 | 0.0118 |
| M3 | 0.0303 | 0.0072 | 0.0424 | 0.006 | 0.029 | 0.0165 | 0.0242 | 0.0066 | 0.021 | 0.0081 | 0.037 | 0.0118 |
| M4 | 0.0067 | 0.0072 | 0.0075 | 0.006 | 0.007 | 0.0165 | 0.0064 | 0.0066 | 0.006 | 0.0081 | 0.007 | 0.0118 |
| M5 | 0.0009 | 0.0072 | 0.0014 | 0.006 | 0.0008 | 0.0165 | 0.0012 | 0.0066 | 0.001 | 0.0081 | 0.001 | 0.0118 |
| **CIT 459** | | | | | | | | | | | | |
| M0 | 0.3039 | 0.0092 | 0.2524 | 0.0074 | 0.3221 | 0.0126 | 0.2455 | 0.0064 | 0.308 | 0.0102 | 0.251 | 0.0074 |
| M1 | 0.1583 | 0.0092 | 0.1359 | 0.0074 | 0.1491 | 0.0126 | 0.1417 | 0.0064 | 0.154 | 0.0102 | 0.153 | 0.0074 |
| M2 | 0.1427 | 0.0092 | 0.1306 | 0.0074 | 0.1292 | 0.0126 | 0.1322 | 0.0064 | 0.133 | 0.0102 | 0.135 | 0.0074 |
| M3 | 0.0874 | 0.0092 | 0.09 | 0.0074 | 0.0895 | 0.0126 | 0.0872 | 0.0064 | 0.092 | 0.0102 | 0.093 | 0.0074 |
| M4 | 0.199 | 0.0092 | 0.1845 | 0.0074 | 0.1889 | 0.0126 | 0.1649 | 0.0064 | 0.185 | 0.0102 | 0.165 | 0.0074 |

| Cell Line | MCF 10A | | | | MCF7 | | | | MDA-MB-231 | | | |
|---|---|---|---|---|---|---|---|---|---|---|---|---|
| Condition | Control | | High-Lactate | | Control | | High-Lactate | | Control | | High-Lactate | |
| | MID | SE | MID | SE | MID | SE | MID | SE | MID | SE | MID | SE |
| M5 | 0.068 | 0.0092 | 0.1359 | 0.0074 | 0.0795 | 0.0126 | 0.1417 | 0.0064 | 0.082 | 0.0102 | 0.131 | 0.0074 |
| M6 | 0.0291 | 0.0092 | 0.0487 | 0.0074 | 0.0298 | 0.0126 | 0.0614 | 0.0064 | 0.034 | 0.0102 | 0.05 | 0.0074 |
| M7 | 0.0087 | 0.0092 | 0.0156 | 0.0074 | 0.0089 | 0.0126 | 0.0189 | 0.0064 | 0.009 | 0.0102 | 0.016 | 0.0074 |
| M8 | 0.0029 | 0.0092 | 0.0066 | 0.0074 | 0.003 | 0.0126 | 0.0066 | 0.0064 | 0.003 | 0.0102 | 0.007 | 0.0074 |
| **AKG 346** | | | | | | | | | | | | |
| M0 | 0.2076 | 0.006 | 0.1916 | 0.006 | 0.2326 | 0.0128 | 0.2037 | 0.007 | 0.207 | 0.0102 | 0.221 | 0.006 |
| M1 | 0.1023 | 0.006 | 0.0993 | 0.006 | 0.091 | 0.0128 | 0.1034 | 0.007 | 0.094 | 0.0102 | 0.106 | 0.006 |
| M2 | 0.0789 | 0.006 | 0.0725 | 0.006 | 0.0808 | 0.0128 | 0.0755 | 0.007 | 0.08 | 0.0102 | 0.074 | 0.006 |
| M3 | 0.1189 | 0.006 | 0.139 | 0.006 | 0.1163 | 0.0128 | 0.1354 | 0.007 | 0.113 | 0.0102 | 0.129 | 0.006 |
| M4 | 0.0448 | 0.006 | 0.0477 | 0.006 | 0.0404 | 0.0128 | 0.0507 | 0.007 | 0.043 | 0.0102 | 0.046 | 0.006 |
| M5 | 0.3402 | 0.006 | 0.3455 | 0.006 | 0.3276 | 0.0128 | 0.3256 | 0.007 | 0.358 | 0.0102 | 0.318 | 0.006 |
| M6 | 0.078 | 0.006 | 0.0745 | 0.006 | 0.0811 | 0.0128 | 0.0744 | 0.007 | 0.075 | 0.0102 | 0.076 | 0.006 |
| M7 | 0.0292 | 0.006 | 0.0301 | 0.006 | 0.0303 | 0.0128 | 0.0313 | 0.007 | 0.028 | 0.0102 | 0.031 | 0.006 |
| **GLU 432** | | | | | | | | | | | | |
| M0 | 0.1701 | 0.0082 | 0.17 | 0.006 | 0.1878 | 0.009 | 0.1425 | 0.0074 | 0.175 | 0.0097 | 0.184 | 0.0065 |
| M1 | 0.0977 | 0.0082 | 0.098 | 0.006 | 0.0995 | 0.009 | 0.0855 | 0.0074 | 0.098 | 0.0097 | 0.108 | 0.0065 |
| M2 | 0.0791 | 0.0082 | 0.076 | 0.006 | 0.0832 | 0.009 | 0.0617 | 0.0074 | 0.082 | 0.0097 | 0.076 | 0.0065 |
| M3 | 0.1109 | 0.0082 | 0.123 | 0.006 | 0.1117 | 0.009 | 0.1007 | 0.0074 | 0.109 | 0.0097 | 0.121 | 0.0065 |
| M4 | 0.0521 | 0.0082 | 0.057 | 0.006 | 0.0508 | 0.009 | 0.0541 | 0.0074 | 0.055 | 0.0097 | 0.056 | 0.0065 |
| M5 | 0.3353 | 0.0082 | 0.3251 | 0.006 | 0.3147 | 0.009 | 0.3751 | 0.0074 | 0.328 | 0.0097 | 0.308 | 0.0065 |
| M6 | 0.1049 | 0.0082 | 0.101 | 0.006 | 0.1015 | 0.009 | 0.1235 | 0.0074 | 0.104 | 0.0097 | 0.099 | 0.0065 |
| M7 | 0.0501 | 0.0082 | 0.05 | 0.006 | 0.0508 | 0.009 | 0.057 | 0.0074 | 0.049 | 0.0097 | 0.049 | 0.0065 |
| **GLN 431** | | | | | | | | | | | | |
| M0 | 0.027 | 0.0121 | 0.0289 | 0.006 | 0.0209 | 0.0184 | 0.0317 | 0.006 | 0.024 | 0.0086 | 0.033 | 0.0073 |
| M1 | 0.0108 | 0.0121 | 0.0108 | 0.006 | 0.0105 | 0.0184 | 0.0123 | 0.006 | 0.012 | 0.0086 | 0.012 | 0.0073 |
| M2 | 0.0049 | 0.0121 | 0.0049 | 0.006 | 0.0063 | 0.0184 | 0.0058 | 0.006 | 0.006 | 0.0086 | 0.006 | 0.0073 |
| M3 | 0.0023 | 0.0121 | 0.0023 | 0.006 | 0.0032 | 0.0184 | 0.002 | 0.006 | 0.003 | 0.0086 | 0.002 | 0.0073 |
| M4 | 0.028 | 0.0121 | 0.0299 | 0.006 | 0.0214 | 0.0184 | 0.0324 | 0.006 | 0.02 | 0.0086 | 0.031 | 0.0073 |
| M5 | 0.6252 | 0.0121 | 0.6232 | 0.006 | 0.6216 | 0.0184 | 0.6239 | 0.006 | 0.638 | 0.0086 | 0.622 | 0.0073 |
| M6 | 0.2048 | 0.0121 | 0.2033 | 0.006 | 0.2107 | 0.0184 | 0.2013 | 0.006 | 0.198 | 0.0086 | 0.203 | 0.0073 |
| M7 | 0.097 | 0.0121 | 0.0969 | 0.006 | 0.1054 | 0.0184 | 0.0906 | 0.006 | 0.099 | 0.0086 | 0.091 | 0.0073 |
| **PRO 258** | | | | | | | | | | | | |
| M0 | 0.2022 | 0.0075 | 0.2024 | 0.006 | 0.2245 | 0.0126 | 0.1684 | 0.0061 | 0.212 | 0.0123 | 0.22 | 0.0062 |

| Cell Line | MCF 10A | | | | MCF7 | | | | MDA-MB-231 | | | |
|---|---|---|---|---|---|---|---|---|---|---|---|---|
| Condition | Control | | High-Lactate | | Control | | High-Lactate | | Control | | High-Lactate | |
| | MID | SE | MID | SE | MID | SE | MID | SE | MID | SE | MID | SE |
| M1 | 0.1085 | 0.0075 | 0.1136 | 0.006 | 0.102 | 0.0126 | 0.0982 | 0.0061 | 0.106 | 0.0123 | 0.118 | 0.0062 |
| M2 | 0.1497 | 0.0075 | 0.161 | 0.006 | 0.1531 | 0.0126 | 0.131 | 0.0061 | 0.155 | 0.0123 | 0.155 | 0.0062 |
| M3 | 0.0493 | 0.0075 | 0.0494 | 0.006 | 0.051 | 0.0126 | 0.0505 | 0.0061 | 0.053 | 0.0123 | 0.05 | 0.0062 |
| M4 | 0.3818 | 0.0075 | 0.3703 | 0.006 | 0.3571 | 0.0126 | 0.4303 | 0.0061 | 0.372 | 0.0123 | 0.353 | 0.0062 |
| M5 | 0.0784 | 0.0075 | 0.0731 | 0.006 | 0.0816 | 0.0126 | 0.0842 | 0.0061 | 0.071 | 0.0123 | 0.073 | 0.0062 |
| M6 | 0.0301 | 0.0075 | 0.0302 | 0.006 | 0.0306 | 0.0126 | 0.0374 | 0.0061 | 0.031 | 0.0123 | 0.03 | 0.0061 |
| **SUCC 289** | | | | | | | | | | | | |
| M0 | 0.3317 | 0.006 | 0.3135 | 0.0074 | 0.3542 | 0.015 | 0.3304 | 0.0126 | 0.33 | 0.0136 | 0.358 | 0.0176 |
| M1 | 0.1275 | 0.006 | 0.1302 | 0.0074 | 0.1302 | 0.015 | 0.1369 | 0.0126 | 0.125 | 0.0136 | 0.136 | 0.0176 |
| M2 | 0.1444 | 0.006 | 0.1511 | 0.0074 | 0.1354 | 0.015 | 0.1479 | 0.0126 | 0.131 | 0.0136 | 0.143 | 0.0176 |
| M3 | 0.0428 | 0.006 | 0.0504 | 0.0074 | 0.0417 | 0.015 | 0.0484 | 0.0126 | 0.04 | 0.0136 | 0.048 | 0.0176 |
| M4 | 0.2739 | 0.006 | 0.2803 | 0.0074 | 0.2604 | 0.015 | 0.2647 | 0.0126 | 0.294 | 0.0136 | 0.245 | 0.0176 |
| M5 | 0.0598 | 0.006 | 0.0544 | 0.0074 | 0.0573 | 0.015 | 0.0523 | 0.0126 | 0.057 | 0.0136 | 0.051 | 0.0176 |
| M6 | 0.0199 | 0.006 | 0.0201 | 0.0074 | 0.0208 | 0.015 | 0.0194 | 0.0126 | 0.023 | 0.0136 | 0.019 | 0.0176 |
| **FUM 287** | | | | | | | | | | | | |
| M0 | 0.3642 | 0.0093 | 0.3432 | 0.0083 | 0.3769 | 0.0145 | 0.3564 | 0.0082 | 0.378 | 0.0124 | 0.37 | 0.0122 |
| M1 | 0.1332 | 0.0093 | 0.132 | 0.0083 | 0.1296 | 0.0145 | 0.1391 | 0.0082 | 0.131 | 0.0124 | 0.141 | 0.0122 |
| M2 | 0.1414 | 0.0093 | 0.1452 | 0.0083 | 0.1396 | 0.0145 | 0.1434 | 0.0082 | 0.138 | 0.0124 | 0.141 | 0.0122 |
| M3 | 0.0407 | 0.0093 | 0.0528 | 0.0083 | 0.0389 | 0.0145 | 0.0489 | 0.0082 | 0.039 | 0.0124 | 0.053 | 0.0122 |
| M4 | 0.2543 | 0.0093 | 0.2578 | 0.0083 | 0.2443 | 0.0145 | 0.2447 | 0.0082 | 0.25 | 0.0124 | 0.229 | 0.0122 |
| M5 | 0.0458 | 0.0093 | 0.0504 | 0.0083 | 0.0469 | 0.0145 | 0.0492 | 0.0082 | 0.045 | 0.0124 | 0.049 | 0.0122 |
| M6 | 0.0203 | 0.0093 | 0.0187 | 0.0083 | 0.0239 | 0.0145 | 0.0182 | 0.0082 | 0.02 | 0.0124 | 0.018 | 0.0122 |
| **MAL 419** | | | | | | | | | | | | |
| M0 | 0.3155 | 0.0099 | 0.2949 | 0.005 | 0.3287 | 0.0166 | 0.31 | 0.009 | 0.319 | 0.0101 | 0.323 | 0.0092 |
| M1 | 0.1555 | 0.0099 | 0.1523 | 0.005 | 0.1524 | 0.0166 | 0.157 | 0.009 | 0.147 | 0.0101 | 0.16 | 0.0092 |
| M2 | 0.143 | 0.0099 | 0.1499 | 0.005 | 0.1394 | 0.0166 | 0.1525 | 0.009 | 0.142 | 0.0101 | 0.15 | 0.0092 |
| M3 | 0.0715 | 0.0099 | 0.0688 | 0.005 | 0.0797 | 0.0166 | 0.062 | 0.009 | 0.076 | 0.0101 | 0.069 | 0.0092 |
| M4 | 0.2145 | 0.0099 | 0.2359 | 0.005 | 0.2052 | 0.0166 | 0.224 | 0.009 | 0.218 | 0.0101 | 0.205 | 0.0092 |
| M5 | 0.0724 | 0.0099 | 0.0688 | 0.005 | 0.0647 | 0.0166 | 0.0647 | 0.009 | 0.065 | 0.0101 | 0.064 | 0.0092 |
| M6 | 0.0277 | 0.0099 | 0.0294 | 0.005 | 0.0299 | 0.0166 | 0.0299 | 0.009 | 0.033 | 0.0101 | 0.029 | 0.0092 |
| **ASP 418** | | | | | | | | | | | | |
| M0 | 0.4362 | 0.006 | 0.4966 | 0.0111 | 0.4602 | 0.011 | 0.3267 | 0.009 | 0.46 | 0.0142 | 0.498 | 0.0121 |
| M1 | 0.1819 | 0.006 | 0.1978 | 0.0111 | 0.1902 | 0.011 | 0.1584 | 0.009 | 0.19 | 0.0142 | 0.198 | 0.0121 |
| M2 | 0.1309 | 0.006 | 0.1233 | 0.0111 | 0.1231 | 0.011 | 0.1484 | 0.009 | 0.123 | 0.0142 | 0.124 | 0.0121 |

| Cell Line | MCF 10A | | | | MCF7 | | | | MDA-MB-231 | | | |
|---|---|---|---|---|---|---|---|---|---|---|---|---|
| Condition | Control | | High-Lactate | | Control | | High-Lactate | | Control | | High-Lactate | |
| | MID | SE | MID | SE | MID | SE | MID | SE | MID | SE | MID | SE |
| M3 | 0.0553 | 0.006 | 0.0393 | 0.0111 | 0.0501 | 0.011 | 0.0587 | 0.009 | 0.056 | 0.0142 | 0.044 | 0.0121 |
| M4 | 0.1362 | 0.006 | 0.0988 | 0.0111 | 0.1202 | 0.011 | 0.2082 | 0.009 | 0.119 | 0.0142 | 0.093 | 0.0121 |
| M5 | 0.0426 | 0.006 | 0.0302 | 0.0111 | 0.0401 | 0.011 | 0.0664 | 0.009 | 0.037 | 0.0142 | 0.03 | 0.0121 |
| M6 | 0.017 | 0.006 | 0.014 | 0.0111 | 0.016 | 0.011 | 0.0332 | 0.009 | 0.016 | 0.0142 | 0.014 | 0.0121 |

**Table G.** Measured intracellular MIDs from [U-$^{13}$C] lactate tracer for the three breast cell lines for the control and high-lactate conditions with mean and standard errors. MIDs shown below have not been corrected for natural abundance. A standard error of 0.006 represents the lower limit error cutoff reported for most instruments [1, 2], which was applied even when the biological replicates had observed lower standard error.

| Cell Line | MCF 10A | | | | MCF7 | | | | MDA-MB-231 | | | |
|---|---|---|---|---|---|---|---|---|---|---|---|---|
| Condition | Control | | High-Lactate | | Control | | High-Lactate | | Control | | High-Lactate | |
| | MID | SE | MID | SE | MID | SE | MID | SE | MID | SE | MID | SE |
| PYR 174 | | | | | | | | | | | | |
| M0 | n/a | n/a | 0.8219 | 0.0065 | n/a | n/a | 0.8166 | 0.0066 | n/a | n/a | 0.8172 | 0.006 |
| M1 | n/a | n/a | 0.1004 | 0.0065 | n/a | n/a | 0.1069 | 0.0066 | n/a | n/a | 0.1034 | 0.006 |
| M2 | n/a | n/a | 0.0404 | 0.0065 | n/a | n/a | 0.0389 | 0.0066 | n/a | n/a | 0.0384 | 0.006 |
| M3 | n/a | n/a | 0.0331 | 0.0065 | n/a | n/a | 0.0339 | 0.0066 | n/a | n/a | 0.0374 | 0.006 |
| M4 | n/a | n/a | 0.0031 | 0.0065 | n/a | n/a | 0.002 | 0.0066 | n/a | n/a | 0.0026 | 0.006 |
| M5 | n/a | n/a | 0.0008 | 0.0065 | n/a | n/a | 0.0014 | 0.0066 | n/a | n/a | 0.001 | 0.006 |
| ALA 260 | | | | | | | | | | | | |
| M0 | n/a | n/a | 0.7268 | 0.0088 | n/a | n/a | 0.7236 | 0.006 | n/a | n/a | 0.7232 | 0.0072 |
| M1 | n/a | n/a | 0.1622 | 0.0088 | n/a | n/a | 0.1616 | 0.006 | n/a | n/a | 0.1599 | 0.0072 |
| M2 | n/a | n/a | 0.0706 | 0.0088 | n/a | n/a | 0.0663 | 0.006 | n/a | n/a | 0.0679 | 0.0072 |
| M3 | n/a | n/a | 0.0313 | 0.0088 | n/a | n/a | 0.0391 | 0.006 | n/a | n/a | 0.0398 | 0.0072 |
| M4 | n/a | n/a | 0.0071 | 0.0088 | n/a | n/a | 0.0069 | 0.006 | n/a | n/a | 0.0068 | 0.0072 |
| M5 | n/a | n/a | 0.0019 | 0.0088 | n/a | n/a | 0.0024 | 0.006 | n/a | n/a | 0.0022 | 0.0072 |
| LAC 261 | | | | | | | | | | | | |
| M0 | n/a | n/a | 0.7201 | 0.006 | n/a | n/a | 0.6884 | 0.0088 | n/a | n/a | 0.7146 | 0.0078 |
| M1 | n/a | n/a | 0.1623 | 0.006 | n/a | n/a | 0.158 | 0.0088 | n/a | n/a | 0.1623 | 0.0078 |
| M2 | n/a | n/a | 0.071 | 0.006 | n/a | n/a | 0.0699 | 0.0088 | n/a | n/a | 0.0689 | 0.0078 |
| M3 | n/a | n/a | 0.0364 | 0.006 | n/a | n/a | 0.0703 | 0.0088 | n/a | n/a | 0.0435 | 0.0078 |
| M4 | n/a | n/a | 0.008 | 0.006 | n/a | n/a | 0.0098 | 0.0088 | n/a | n/a | 0.008 | 0.0078 |
| M5 | n/a | n/a | 0.0021 | 0.006 | n/a | n/a | 0.0033 | 0.0088 | n/a | n/a | 0.0022 | 0.0078 |
| CIT 459 | | | | | | | | | | | | |
| M0 | n/a | n/a | 0.6202 | 0.0086 | n/a | n/a | 0.6157 | 0.0114 | n/a | n/a | 0.6182 | 0.006 |
| M1 | n/a | n/a | 0.2303 | 0.0086 | n/a | n/a | 0.2354 | 0.0114 | n/a | n/a | 0.2331 | 0.006 |
| M2 | n/a | n/a | 0.1101 | 0.0086 | n/a | n/a | 0.1106 | 0.0114 | n/a | n/a | 0.1107 | 0.006 |
| M3 | n/a | n/a | 0.028 | 0.0086 | n/a | n/a | 0.0272 | 0.0114 | n/a | n/a | 0.027 | 0.006 |
| M4 | n/a | n/a | 0.0073 | 0.0086 | n/a | n/a | 0.007 | 0.0114 | n/a | n/a | 0.0066 | 0.006 |

| Cell Line | MCF 10A | | | | MCF7 | | | | MDA-MB-231 | | | |
|---|---|---|---|---|---|---|---|---|---|---|---|---|
| Condition | Control | | High-Lactate | | Control | | High-Lactate | | Control | | High-Lactate | |
| | MID | SE | MID | SE | MID | SE | MID | SE | MID | SE | MID | SE |
| M5 | n/a | n/a | 0.0013 | 0.0086 | n/a | n/a | 0.0013 | 0.0114 | n/a | n/a | 0.0014 | 0.006 |
| M6 | n/a | n/a | 0.0011 | 0.0086 | n/a | n/a | 0.0011 | 0.0114 | n/a | n/a | 0.0011 | 0.006 |
| M7 | n/a | n/a | 0.0008 | 0.0086 | n/a | n/a | 0.0009 | 0.0114 | n/a | n/a | 0.0009 | 0.006 |
| M8 | n/a | n/a | 0.0008 | 0.0086 | n/a | n/a | 0.0008 | 0.0114 | n/a | n/a | 0.0008 | 0.006 |
| **AKG 346** | | | | | | | | | | | | |
| M0 | n/a | n/a | 0.7131 | 0.009 | n/a | n/a | 0.7145 | 0.006 | n/a | n/a | 0.7147 | 0.006 |
| M1 | n/a | n/a | 0.1882 | 0.009 | n/a | n/a | 0.1898 | 0.006 | n/a | n/a | 0.1884 | 0.006 |
| M2 | n/a | n/a | 0.0791 | 0.009 | n/a | n/a | 0.0767 | 0.006 | n/a | n/a | 0.0774 | 0.006 |
| M3 | n/a | n/a | 0.0139 | 0.009 | n/a | n/a | 0.0127 | 0.006 | n/a | n/a | 0.0138 | 0.006 |
| M4 | n/a | n/a | 0.0029 | 0.009 | n/a | n/a | 0.0032 | 0.006 | n/a | n/a | 0.0029 | 0.006 |
| M5 | n/a | n/a | 0.0011 | 0.009 | n/a | n/a | 0.0011 | 0.006 | n/a | n/a | 0.0011 | 0.006 |
| M6 | n/a | n/a | 0.0009 | 0.009 | n/a | n/a | 0.001 | 0.006 | n/a | n/a | 0.0007 | 0.006 |
| M7 | n/a | n/a | 0.0006 | 0.009 | n/a | n/a | 0.0008 | 0.006 | n/a | n/a | 0.0007 | 0.006 |
| **GLU 432** | | | | | | | | | | | | |
| M0 | n/a | n/a | 0.6183 | 0.0125 | n/a | n/a | 0.6257 | 0.006 | n/a | n/a | 0.6248 | 0.006 |
| M1 | n/a | n/a | 0.2354 | 0.0125 | n/a | n/a | 0.2304 | 0.006 | n/a | n/a | 0.2325 | 0.006 |
| M2 | n/a | n/a | 0.1078 | 0.0125 | n/a | n/a | 0.108 | 0.006 | n/a | n/a | 0.108 | 0.006 |
| M3 | n/a | n/a | 0.0293 | 0.0125 | n/a | n/a | 0.0269 | 0.006 | n/a | n/a | 0.0263 | 0.006 |
| M4 | n/a | n/a | 0.0058 | 0.0125 | n/a | n/a | 0.0061 | 0.006 | n/a | n/a | 0.0056 | 0.006 |
| M5 | n/a | n/a | 0.0019 | 0.0125 | n/a | n/a | 0.0016 | 0.006 | n/a | n/a | 0.0014 | 0.006 |
| M6 | n/a | n/a | 0.0009 | 0.0125 | n/a | n/a | 0.0009 | 0.006 | n/a | n/a | 0.0009 | 0.006 |
| M7 | n/a | n/a | 0.0001 | 0.0125 | n/a | n/a | 0.0003 | 0.006 | n/a | n/a | 0.0001 | 0.006 |
| **GLN 431** | | | | | | | | | | | | |
| M0 | n/a | n/a | 0.6261 | 0.006 | n/a | n/a | 0.6253 | 0.0088 | n/a | n/a | 0.624 | 0.006 |
| M1 | n/a | n/a | 0.2323 | 0.006 | n/a | n/a | 0.2344 | 0.0088 | n/a | n/a | 0.2342 | 0.006 |
| M2 | n/a | n/a | 0.108 | 0.006 | n/a | n/a | 0.1065 | 0.0088 | n/a | n/a | 0.1073 | 0.006 |
| M3 | n/a | n/a | 0.0251 | 0.006 | n/a | n/a | 0.0259 | 0.0088 | n/a | n/a | 0.0262 | 0.006 |
| M4 | n/a | n/a | 0.006 | 0.006 | n/a | n/a | 0.0057 | 0.0088 | n/a | n/a | 0.0059 | 0.006 |
| M5 | n/a | n/a | 0.0012 | 0.006 | n/a | n/a | 0.0012 | 0.0088 | n/a | n/a | 0.0013 | 0.006 |
| M6 | n/a | n/a | 0.0008 | 0.006 | n/a | n/a | 0.0008 | 0.0088 | n/a | n/a | 0.0008 | 0.006 |
| M7 | n/a | n/a | 0.0001 | 0.006 | n/a | n/a | 0.0001 | 0.0088 | n/a | n/a | 0.0001 | 0.006 |
| **PRO 258** | | | | | | | | | | | | |
| M0 | n/a | n/a | 0.7343 | 0.0091 | n/a | n/a | 0.7378 | 0.0048 | n/a | n/a | 0.7373 | 0.006 |
| M1 | n/a | n/a | 0.1811 | 0.0091 | n/a | n/a | 0.1802 | 0.0048 | n/a | n/a | 0.1778 | 0.006 |

| Cell Line | MCF 10A | | | | MCF7 | | | | MDA-MB-231 | | | |
|---|---|---|---|---|---|---|---|---|---|---|---|---|
| Condition | Control | | High-Lactate | | Control | | High-Lactate | | Control | | High-Lactate | |
| | MID | SE | MID | SE | MID | SE | MID | SE | MID | SE | MID | SE |
| M2 | n/a | n/a | 0.0704 | 0.0091 | n/a | n/a | 0.0704 | 0.0048 | n/a | n/a | 0.0698 | 0.006 |
| M3 | n/a | n/a | 0.0101 | 0.0091 | n/a | n/a | 0.0082 | 0.0048 | n/a | n/a | 0.0113 | 0.006 |
| M4 | n/a | n/a | 0.003 | 0.0091 | n/a | n/a | 0.0023 | 0.0048 | n/a | n/a | 0.0025 | 0.006 |
| M5 | n/a | n/a | 0.0007 | 0.0091 | n/a | n/a | 0.0008 | 0.0048 | n/a | n/a | 0.0007 | 0.006 |
| M6 | n/a | n/a | 0.0003 | 0.0091 | n/a | n/a | 0.0003 | 0.0048 | n/a | n/a | 0.0004 | 0.006 |
| **SUCC 289** | | | | | | | | | | | | |
| M0 | n/a | n/a | 0.7346 | 0.0097 | n/a | n/a | 0.7368 | 0.006 | n/a | n/a | 0.735 | 0.006 |
| M1 | n/a | n/a | 0.1736 | 0.0097 | n/a | n/a | 0.1743 | 0.006 | n/a | n/a | 0.1754 | 0.006 |
| M2 | n/a | n/a | 0.0763 | 0.0097 | n/a | n/a | 0.0746 | 0.006 | n/a | n/a | 0.0738 | 0.006 |
| M3 | n/a | n/a | 0.0108 | 0.0097 | n/a | n/a | 0.0109 | 0.006 | n/a | n/a | 0.0114 | 0.006 |
| M4 | n/a | n/a | 0.0029 | 0.0097 | n/a | n/a | 0.0026 | 0.006 | n/a | n/a | 0.0029 | 0.006 |
| M5 | n/a | n/a | 0.0009 | 0.0097 | n/a | n/a | 0.0006 | 0.006 | n/a | n/a | 0.0009 | 0.006 |
| M6 | n/a | n/a | 0.0005 | 0.0097 | n/a | n/a | 0.0002 | 0.006 | n/a | n/a | 0.0005 | 0.006 |
| **FUM 287** | | | | | | | | | | | | |
| M0 | n/a | n/a | 0.7349 | 0.006 | n/a | n/a | 0.7345 | 0.0061 | n/a | n/a | 0.7357 | 0.0101 |
| M1 | n/a | n/a | 0.1739 | 0.006 | n/a | n/a | 0.1756 | 0.0061 | n/a | n/a | 0.1751 | 0.0101 |
| M2 | n/a | n/a | 0.075 | 0.006 | n/a | n/a | 0.0735 | 0.0061 | n/a | n/a | 0.0737 | 0.0101 |
| M3 | n/a | n/a | 0.012 | 0.006 | n/a | n/a | 0.0126 | 0.0061 | n/a | n/a | 0.0119 | 0.0101 |
| M4 | n/a | n/a | 0.0027 | 0.006 | n/a | n/a | 0.0027 | 0.0061 | n/a | n/a | 0.0026 | 0.0101 |
| M5 | n/a | n/a | 0.0009 | 0.006 | n/a | n/a | 0.0005 | 0.0061 | n/a | n/a | 0.0006 | 0.0101 |
| M6 | n/a | n/a | 0.0005 | 0.006 | n/a | n/a | 0.0004 | 0.0061 | n/a | n/a | 0.0003 | 0.0101 |
| **MAL 419** | | | | | | | | | | | | |
| M0 | n/a | n/a | 0.6328 | 0.0123 | n/a | n/a | 0.6325 | 0.0084 | n/a | n/a | 0.6338 | 0.006 |
| M1 | n/a | n/a | 0.227 | 0.0123 | n/a | n/a | 0.2278 | 0.0084 | n/a | n/a | 0.2262 | 0.006 |
| M2 | n/a | n/a | 0.1075 | 0.0123 | n/a | n/a | 0.1074 | 0.0084 | n/a | n/a | 0.1075 | 0.006 |
| M3 | n/a | n/a | 0.0251 | 0.0123 | n/a | n/a | 0.0258 | 0.0084 | n/a | n/a | 0.0251 | 0.006 |
| M4 | n/a | n/a | 0.0055 | 0.0123 | n/a | n/a | 0.0043 | 0.0084 | n/a | n/a | 0.005 | 0.006 |
| M5 | n/a | n/a | 0.0009 | 0.0123 | n/a | n/a | 0.001 | 0.0084 | n/a | n/a | 0.0014 | 0.006 |
| M6 | n/a | n/a | 0.0008 | 0.0123 | n/a | n/a | 0.0009 | 0.0084 | n/a | n/a | 0.0009 | 0.006 |
| **ASP 418** | | | | | | | | | | | | |
| M0 | n/a | n/a | 0.6289 | 0.0149 | n/a | n/a | 0.6312 | 0.0135 | n/a | n/a | 0.6319 | 0.0138 |
| M1 | n/a | n/a | 0.2295 | 0.0149 | n/a | n/a | 0.2295 | 0.0135 | n/a | n/a | 0.2286 | 0.0138 |
| M2 | n/a | n/a | 0.1096 | 0.0149 | n/a | n/a | 0.1067 | 0.0135 | n/a | n/a | 0.107 | 0.0138 |
| M3 | n/a | n/a | 0.0249 | 0.0149 | n/a | n/a | 0.0251 | 0.0135 | n/a | n/a | 0.0253 | 0.0138 |

| Cell Line | MCF 10A | | | | MCF7 | | | | MDA-MB-231 | | | |
|---|---|---|---|---|---|---|---|---|---|---|---|---|
| Condition | Control | | High-Lactate | | Control | | High-Lactate | | Control | | High-Lactate | |
| | MID | SE | MID | SE | MID | SE | MID | SE | MID | SE | MID | SE |
| M4 | n/a | n/a | 0.0052 | 0.0149 | n/a | n/a | 0.005 | 0.0135 | n/a | n/a | 0.005 | 0.0138 |
| M5 | n/a | n/a | 0.0011 | 0.0149 | n/a | n/a | 0.0016 | 0.0135 | n/a | n/a | 0.0013 | 0.0138 |
| M6 | n/a | n/a | 0.0007 | 0.0149 | n/a | n/a | 0.0008 | 0.0135 | n/a | n/a | 0.0007 | 0.0138 |

**Fig B.** Metabolite isotope labeling distribution from [1,2-$^{13}$C] glucose for the MCF 10A, MCF7, and MDA-MB-231 cultures for both control and high-lactate conditions. MIDs shown below have been corrected for natural abundance.

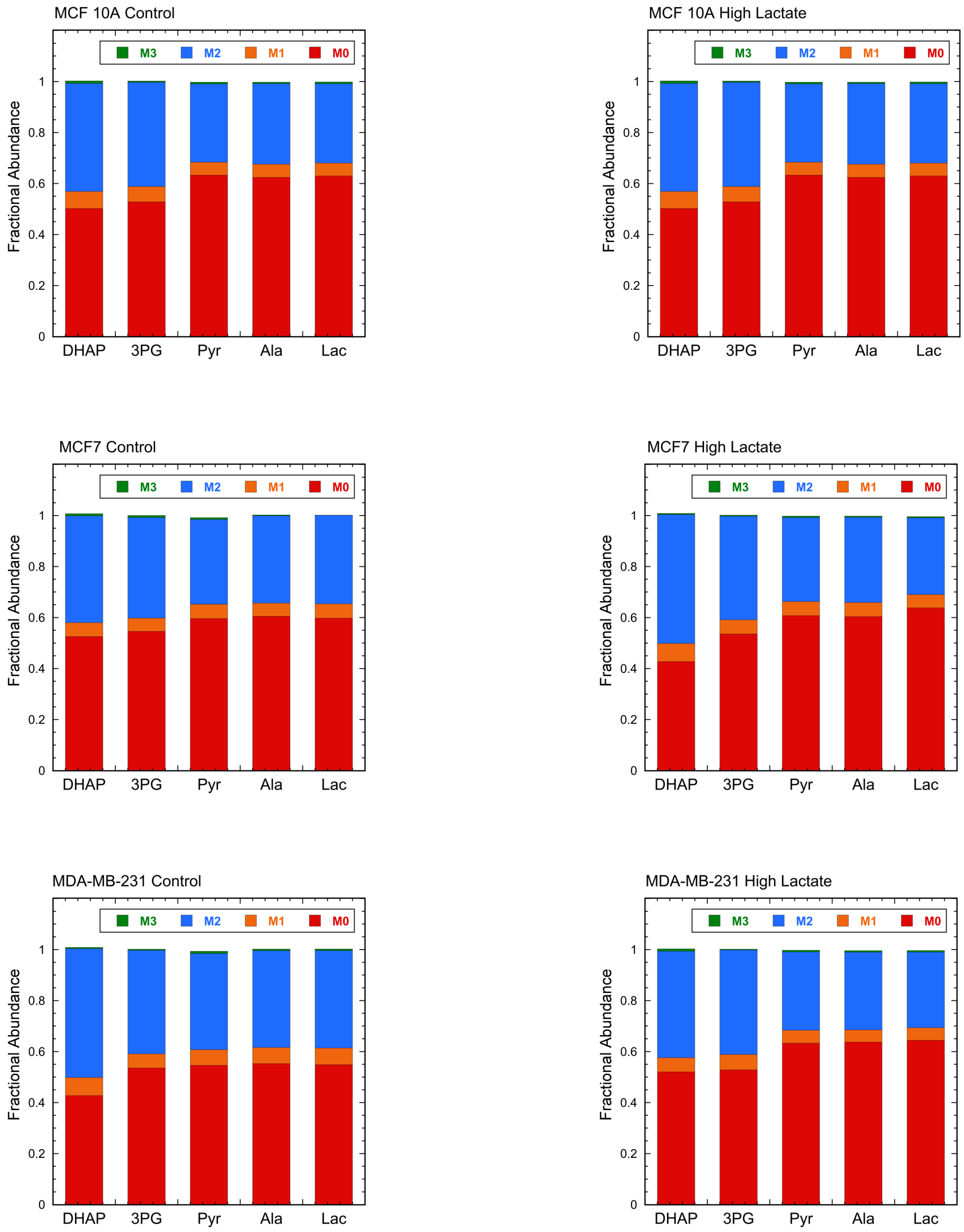

**Fig C.** Metabolite isotope labeling distribution from [U-$^{13}$C] glutamine for the MCF 10A, MCF7, and MDA-MB-231 cultures for both control and high-lactate conditions. MIDs shown below have been corrected for natural abundance.

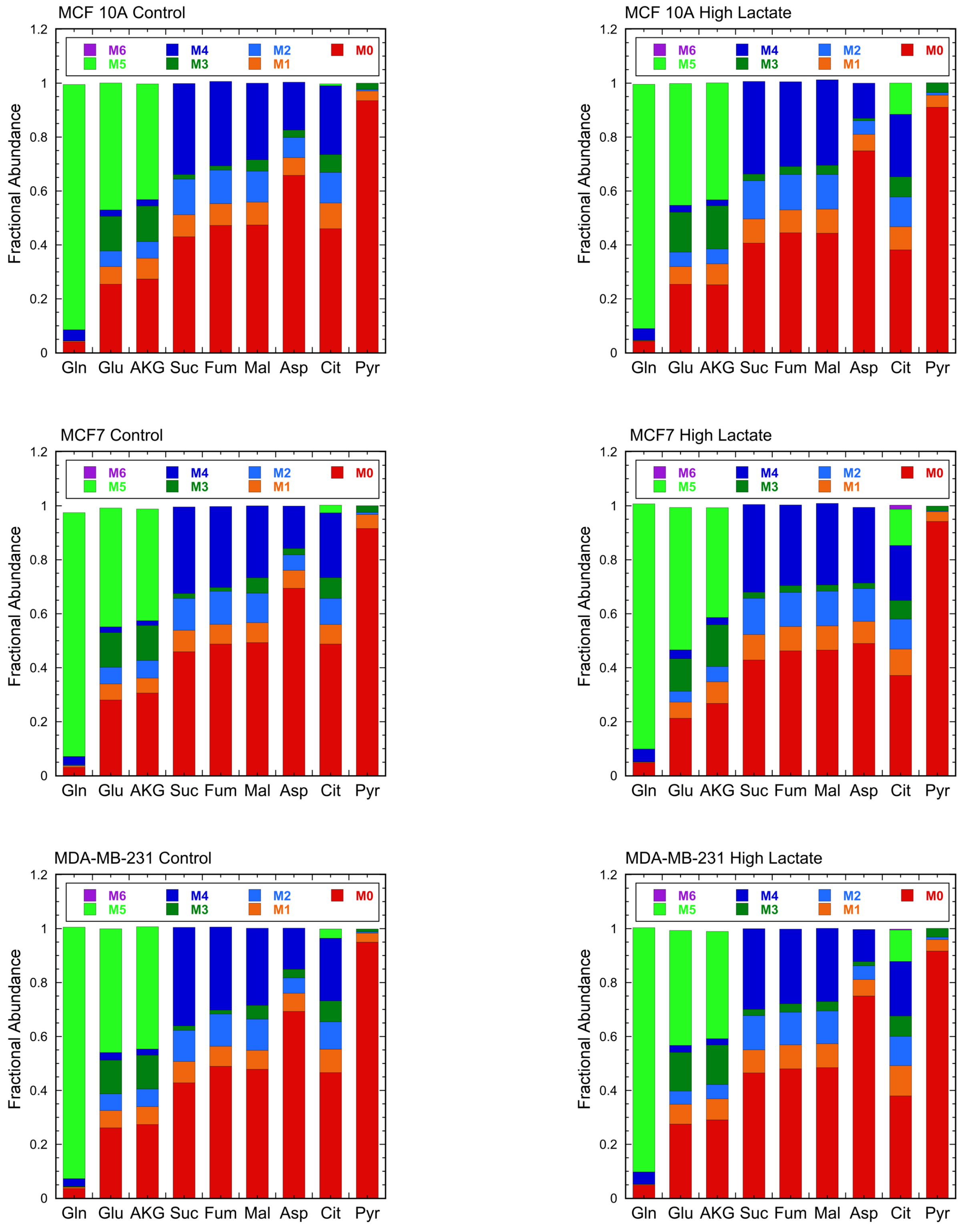

**Fig D.** Metabolic flux maps for MCF 10A predicted from the MFA simulations: A) control and B) high-lactate cultures. The line thicknesses represent the relative fluxes, which are also shown numerically (nmol/$10^6$ cells·h) with standard deviation.

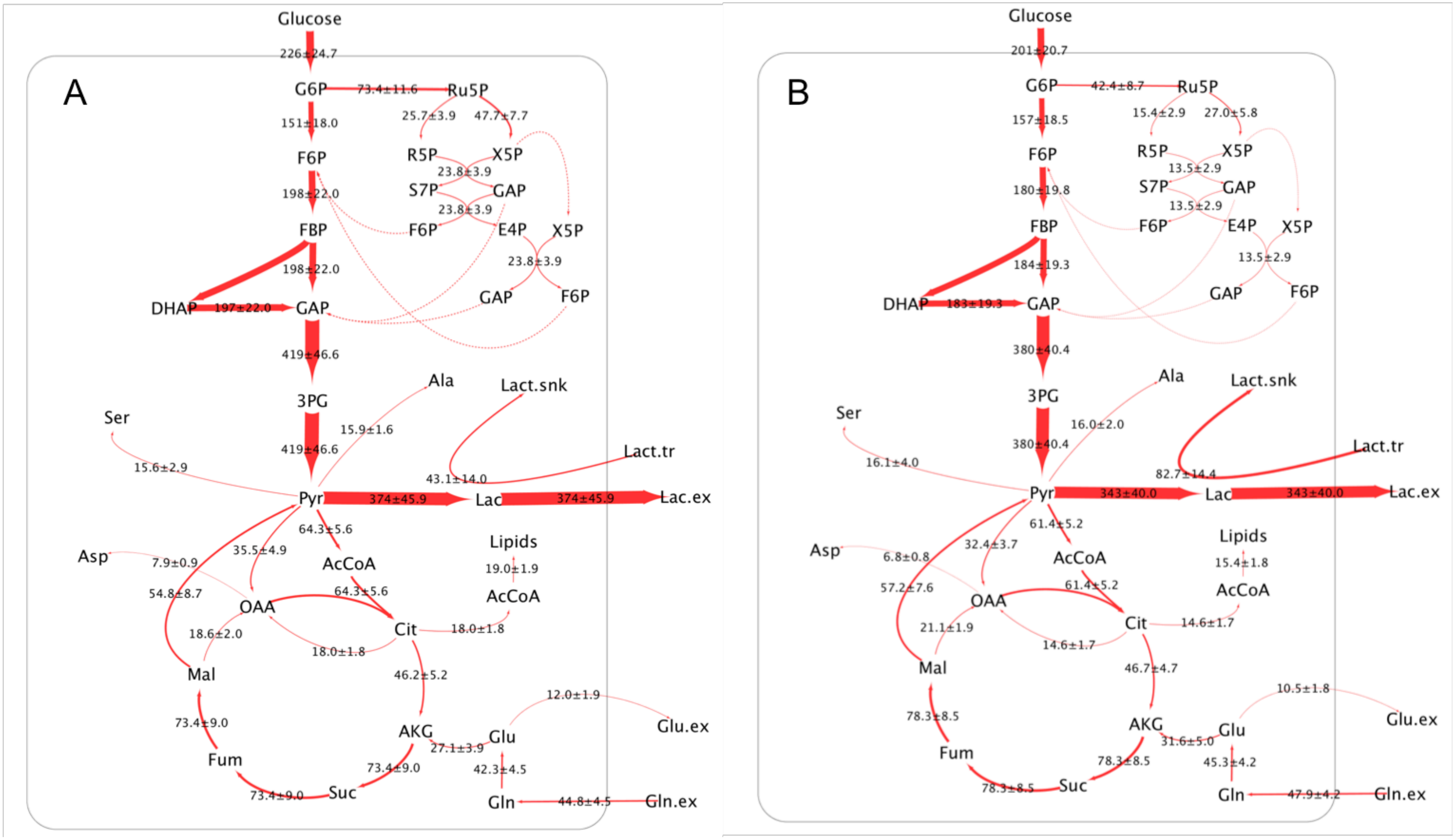

**Fig E.** Metabolic flux maps for MCF7 predicted from the MFA simulations: A) control and B) high-lactate cultures. The line thicknesses represent the relative fluxes, which are also shown numerically (nmol/$10^6$ cells·h) with standard deviations.

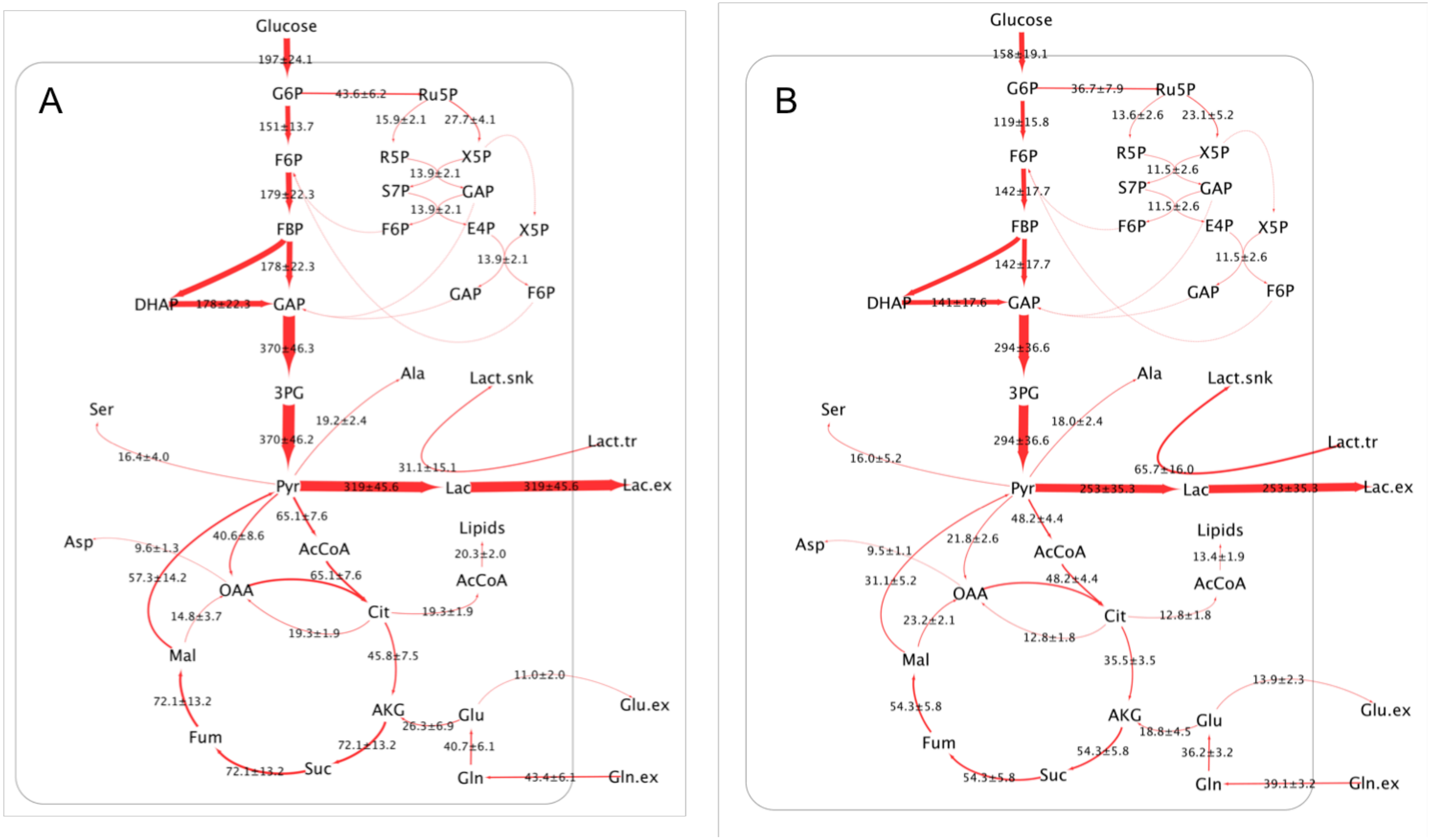

**Fig F.** Metabolic flux maps for MDA-MB-231 predicted from the MFA simulations: A) control and B) high-lactate cultures. The line thicknesses represent the relative fluxes, which are also shown numerically (nmol/$10^6$ cells·h) with standard deviations.

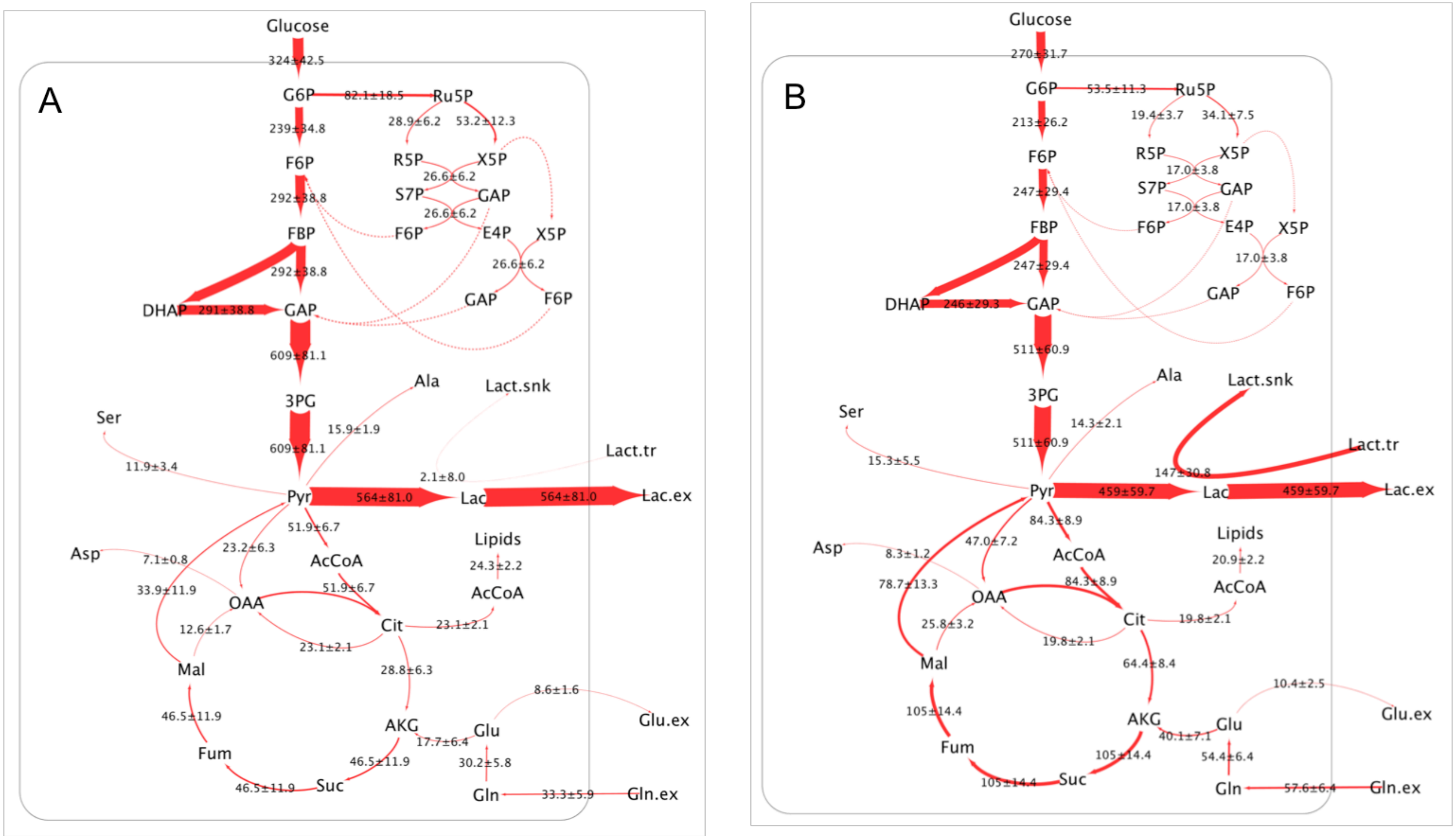

**Table H.** Metabolic flux analysis results for MCF 10A control cultures using [1,2-$^{13}$C] glucose and [U-$^{13}$C] glutamine. The fluxes are shown with 95% confidence intervals. The sum of squared residuals (SSR) was 60.0 with an expected range of 34.0 to 73.8.

| **Reaction** | **Flux** | **95% Confidence Interval** | |
|---|---|---|---|
| | nmol/10$^6$ cells/h | Lower Bound | Upper Bound |
| Gluc.ext -> G6P.c | 226.264 | 179.76 | 276.13 |
| G6P.c <=> F6P.c (net) | 150.514 | 118.53 | 188.56 |
| G6P.c <=> F6P.c (exch) | (>1e4, >1e4) | >10000 | >10000 |
| F6P.c -> FBP.c | 198.197 | 156.94 | 242.61 |
| FBP.c <=> DHAP.c + GAP.c (net) | 198.197 | 156.94 | 242.61 |
| FBP.c <=> DHAP.c + GAP.c (exch) | (0.0, >1e4) | 0 | >10000 |
| DHAP.c <=> GAP.c (net) | 197.247 | 155.99 | 241.66 |
| DHAP.c <=> GAP.c (exch) | 333.237 | 249.63 | 436.75 |
| GAP.c <=> 3PG.c (net) | 419.285 | 331.51 | 513.41 |
| GAP.c <=> 3PG.c (exch) | (0.0, >1e4) | 0 | >10000 |
| 3PG.c -> Pyr.c | 419.285 | 331.51 | 513.41 |
| G6P.c -> CO2 + Ru5P.c | 73.426 | 49.97 | 95.25 |
| Ru5P.c <=> R5P.c (net) | 25.743 | 17.93 | 33.02 |
| Ru5P.c <=> R5P.c (exch) | (>1e4, >1e4) | 0 | >10000 |
| Ru5P.c <=> X5P.c (net) | 47.683 | 32.12 | 62.23 |
| Ru5P.c <=> X5P.c (exch) | (0.0, >1e4) | 0 | >10000 |
| X5P.c + R5P.c <=> GAP.c + S7P.c (net) | 23.841 | 16.06 | 31.11 |
| X5P.c + R5P.c <=> GAP.c + S7P.c (exch) | 1.685 | 0 | 18.81 |
| S7P.c + GAP.c <=> E4P.c + F6P.c (net) | 23.841 | 16.06 | 31.11 |
| S7P.c + GAP.c <=> E4P.c + F6P.c (exch) | (>1e4, >1e4) | 0 | >10000 |
| X5P.c + E4P.c <=> GAP.c + F6P (net) | 23.841 | 16.06 | 31.11 |
| X5P.c + E4P.c <=> GAP.c + F6P (exch) | 0 | 0 | 19.75 |
| Pyr.c <=> Lact.c (net) | 373.984 | 288.09 | 467.14 |
| Pyr.c <=> Lact.c (exch) | (>1000, >1e4) | 351.11 | >10000 |
| Pyr.c -> CO2 + AcCoA.m | 64.337 | 54.81 | 76.6 |
| AcCoA.m + OAC.m -> Cit.m | 64.337 | 54.81 | 76.6 |
| Cit.m <=> AKG.m + CO2 (net) | 46.289 | 37.58 | 58.03 |
| Cit.m <=> AKG.m + CO2 (exch) | 1.786 | 0 | 3.9 |
| AKG.m -> CO2 + Suc.m | 73.402 | 59.19 | 94.47 |
| Suc.m <=> Fum.m (net) | 73.402 | 59.19 | 94.47 |
| Suc.m <=> Fum.m (exch) | 268.25 | 160.72 | 538.33 |
| Fum.m <=> Mal.m (net) | 73.402 | 59.19 | 94.47 |
| Fum.m <=> Mal.m (exch) | (609.3, 928.4) | 297.52 | >10000 |
| Mal.m <=> OAA.m (net) | 18.644 | 14.73 | 22.67 |
| Mal.m <=> OAA.m (exch) | (642.6, 909.8) | 333.54 | >10000 |
| Gln.c -> Glu.c | 42.29 | 34.77 | 52.21 |
| Ser.c <=> Pyr.c (net) | 15.633 | 9.94 | 21.33 |
| Ser.c <=> Pyr.c (exch) | (>1000, >1e4) | 0 | >10000 |
| Gln.ext -> Gln.c | 44.825 | 37.15 | 54.8 |
| Asp.c -> Asp.ext | 4.897 | 3.34 | 6.46 |

| **Reaction** | **Flux** | **95% Confidence Interval** | |
|---|---|---|---|
| | nmol/$10^6$ cells/h | Lower Bound | Upper Bound |
| Ser.ext -> Ser.c | 19.013 | 13.36 | 24.68 |
| Ala.c -> Ala.ext | 10.996 | 8.07 | 13.92 |
| Glu.c -> Glu.ext | 12.007 | 8.32 | 15.7 |
| Lact.c -> Lact.ext | 373.984 | 288.09 | 467.14 |
| Glu.c <=> AKG.m (net) | 3.403 | 0 | 13.46 |
| Glu.c <=> AKG.m (exch) | (0.0, 424.8) | 0 | 660.43 |
| Mal.m -> Pyr.c + CO2 | 54.759 | 41.87 | 75.66 |
| Cit.m -> OAA.m + AcCoA.c | 18.048 | 14.53 | 21.56 |
| Pyr.c + CO2 -> OAA.m | 35.5 | 27.82 | 47.12 |
| 0.95 AcCoA.c + 0.05 DHAP.c -> Lipid | 18.998 | 15.29 | 22.69 |
| Pyr.c + Glu.c <=> Ala.c + AKG.m (net) | 15.856 | 12.77 | 18.93 |
| Pyr.c + Glu.c <=> Ala.c + AKG.m (exch) | 375.738 | 0 | 660.43 |
| OAA.m + Glu.c <=> Asp.c + AKG.m (net) | 7.855 | 6.19 | 9.51 |
| OAA.m + Glu.c <=> Asp.c + AKG.m (exch) | (0.0, 424.8) | 0 | 660.43 |
| 0.23 Ala.c + 0.14 Asp.c + 0.12 Gln.c + 0.15 Glu.c + 0.16 Ser.c + 0.11 G6P.c + 0.09 R5P.c -> Biomass | 21.129 | 17.04 | 25.21 |
| Lact.c + Lact.tr -> Lact.c + Lact.snk | 43.123 | 20.25 | 74.95 |
| Glu.c <=> AKG.m (total) | 27.114 | | |
| Net CO2 production | 276.713 | 226.45 | 342.45 |

**Table I.** Metabolic flux analysis results for MCF 10A high-lactate cultures using [1,2-$^{13}$C] glucose and [U-$^{13}$C] glutamine. The fluxes are shown with 95% confidence intervals. The sum of squared residuals (SSR) was 45.0 with an expected range of 34.8 to 75.0.

| **Reaction** | **Flux** | **95% Confidence Interval** | |
|---|---|---|---|
| | nmol/10$^6$ cells/h | Lower Bound | Upper Bound |
| Gluc.ext -> G6P.c | 201.406 | 162.31 | 243.22 |
| G6P.c <=> F6P.c (net) | 156.651 | 123.84 | 196.17 |
| G6P.c <=> F6P.c (exch) | (>1e4, >1e4) | 0 | >10000 |
| F6P.c -> FBP.c | 183.631 | 146 | 221.22 |
| FBP.c <=> DHAP.c + GAP.c (net) | 183.631 | 146 | 221.22 |
| FBP.c <=> DHAP.c + GAP.c (exch) | (0.0, >1e4) | 0 | >10000 |
| DHAP.c <=> GAP.c (net) | 182.861 | 145.3 | 220.68 |
| DHAP.c <=> GAP.c (exch) | (>1000, >1000) | >1000 | >10000 |
| GAP.c <=> 3PG.c (net) | 379.981 | 302.12 | 459.52 |
| GAP.c <=> 3PG.c (exch) | (0.0, >1e4) | 0 | >10000 |
| 3PG.c -> Pyr.c | 379.981 | 302.12 | 459.52 |
| G6P.c -> CO2 + Ru5P.c | 42.398 | 22.42 | 56.24 |
| Ru5P.c <=> R5P.c (net) | 15.418 | 8.76 | 20.05 |
| Ru5P.c <=> R5P.c (exch) | (>1e4, >1e4) | 0 | >10000 |
| Ru5P.c <=> X5P.c (net) | 26.98 | 13.65 | 36.2 |
| Ru5P.c <=> X5P.c (exch) | (0.0, >1e4) | 0 | >10000 |
| X5P.c + R5P.c <=> GAP.c + S7P.c (net) | 13.49 | 6.83 | 18.1 |
| X5P.c + R5P.c <=> GAP.c + S7P.c (exch) | (>1e4, >1e4) | 0 | >10000 |
| S7P.c + GAP.c <=> E4P.c + F6P.c (net) | 13.49 | 6.83 | 18.1 |
| S7P.c + GAP.c <=> E4P.c + F6P.c (exch) | 3.214 | 0 | 16.9 |
| X5P.c + E4P.c <=> GAP.c + F6P (net) | 13.49 | 6.83 | 18.1 |
| X5P.c + E4P.c <=> GAP.c + F6P (exch) | 0 | 0 | 22.54 |
| Pyr.c <=> Lact.c (net) | 343.45 | 266.56 | 421.74 |
| Pyr.c <=> Lact.c (exch) | (>1e4, >1e4) | >10000 | >10000 |
| Pyr.c -> CO2 + AcCoA.m | 61.356 | 51.64 | 72.08 |
| AcCoA.m + OAC.m -> Cit.m | 61.356 | 51.64 | 72.08 |
| Cit.m <=> AKG.m + CO2 (net) | 46.729 | 38.24 | 56.5 |
| Cit.m <=> AKG.m + CO2 (exch) | 17.296 | 13.73 | 21.52 |
| AKG.m -> CO2 + Suc.m | 78.318 | 62.97 | 96.02 |
| Suc.m <=> Fum.m (net) | 78.318 | 62.97 | 96.02 |
| Suc.m <=> Fum.m (exch) | 277.593 | 177.68 | 476.69 |
| Fum.m <=> Mal.m (net) | 78.318 | 62.97 | 96.02 |
| Fum.m <=> Mal.m (exch) | (>1e4, >1e4) | >10000 | >10000 |
| Mal.m <=> OAA.m (net) | 21.112 | 17.56 | 25 |
| Mal.m <=> OAA.m (exch) | 632.85 | 362.99 | >1000 |
| Gln.c -> Glu.c | 45.321 | 37.65 | 53.99 |
| Ser.c <=> Pyr.c (net) | 16.099 | 8.29 | 23.91 |
| Ser.c <=> Pyr.c (exch) | (>1e4, >1e4) | 0 | >10000 |
| Gln.ext -> Gln.c | 47.892 | 40.16 | 56.58 |

| **Reaction** | **Flux** | **95% Confidence Interval** | |
|---|---|---|---|
| | nmol/$10^6$ cells/h | Lower Bound | Upper Bound |
| Asp.c -> Asp.ext | 3.83 | 2.47 | 5.19 |
| Ser.ext -> Ser.c | 19.527 | 11.74 | 27.31 |
| Ala.c -> Ala.ext | 11.107 | 7.4 | 14.81 |
| Glu.c -> Glu.ext | 10.518 | 7.03 | 14.01 |
| Lact.c -> Lact.ext | 343.45 | 266.56 | 421.74 |
| Glu.c <=> AKG.m (net) | 8.725 | 0.35 | 17.97 |
| Glu.c <=> AKG.m (exch) | (0.0, >1000) | 0 | >1000 |
| Mal.m -> Pyr.c + CO2 | 57.207 | 43.44 | 73.15 |
| Cit.m -> OAA.m + AcCoA.c | 14.627 | 11.33 | 18 |
| Pyr.c + CO2 -> OAA.m | 32.447 | 25.88 | 40.24 |
| 0.95 AcCoA.c + 0.05 DHAP.c -> Lipid | 15.397 | 11.92 | 18.95 |
| Pyr.c + Glu.c <=> Ala.c + AKG.m (net) | 16.034 | 12.22 | 19.85 |
| Pyr.c + Glu.c <=> Ala.c + AKG.m (exch) | (0.0, >1000) | 0 | >1000 |
| OAA.m + Glu.c <=> Asp.c + AKG.m (net) | 6.83 | 5.35 | 8.31 |
| OAA.m + Glu.c <=> Asp.c + AKG.m (exch) | (0.0, >1000) | 0 | >1000 |
| 0.23 Ala.c + 0.14 Asp.c + 0.12 Gln.c + 0.15 Glu.c + 0.16 Ser.c + 0.11 G6P.c + 0.09 R5P.c -> Biomass | 21.425 | 17.34 | 25.51 |
| Lact.c + Lact.tr -> Lact.c + Lact.snk | 82.742 | 56.54 | 112.82 |
| Glu.c <=> AKG.m (total) | 31.589 | | |
| Net CO2 production | 253.56 | 202.62 | 306.3 |

**Table J.** Metabolic flux analysis results for MCF7 control cultures using [1,2-$^{13}$C] glucose and [U-$^{13}$C] glutamine using the biological replicate MIDs for intracellular glutamine, which had a very high standard error. The fluxes are shown with 95% confidence intervals. The sum of squared residuals (SSR) was 30.8 with an expected range of 31.6 to 70.2. **Table S14** has the metabolic flux results for this same culture where the standard error was replaced with 0.6 mol% for one biological replicate. The fluxes are not significantly different between the two simulations.

| Reaction | Flux | 95% Confidence Interval | |
|---|---|---|---|
| | nmol/$10^6$ cells/h | Lower Bound | Upper Bound |
| Gluc.ext -> G6P.c | 197.813 | 150.91 | 244.41 |
| G6P.c <=> F6P.c (net) | 149.733 | 112.58 | 190.06 |
| G6P.c <=> F6P.c (exch) | (>1000, >1e4) | 0 | >10000 |
| F6P.c -> FBP.c | 178.799 | 135.8 | 221.9 |
| FBP.c <=> DHAP.c + GAP.c (net) | 178.799 | 135.8 | 221.9 |
| FBP.c <=> DHAP.c + GAP.c (exch) | (0.0, >1e4) | 0 | >10000 |
| DHAP.c <=> GAP.c (net) | 177.784 | 134.79 | 220.88 |
| DHAP.c <=> GAP.c (exch) | (>1000, >1e4) | >1000 | >10000 |
| GAP.c <=> 3PG.c (net) | 371.116 | 281.09 | 460.47 |
| GAP.c <=> 3PG.c (exch) | (0.0, >1e4) | 0 | >10000 |
| 3PG.c -> Pyr.c | 371.116 | 281.09 | 460.47 |
| G6P.c -> CO2 + Ru5P.c | 45.615 | 24.29 | 67.51 |
| Ru5P.c <=> R5P.c (net) | 16.55 | 9.4 | 23.86 |
| Ru5P.c <=> R5P.c (exch) | (>1e4, >1e4) | 0 | >10000 |
| Ru5P.c <=> X5P.c (net) | 29.066 | 14.82 | 43.66 |
| Ru5P.c <=> X5P.c (exch) | (>1e4, >1e4) | 0 | >10000 |
| X5P.c + R5P.c <=> GAP.c + S7P.c (net) | 14.533 | 7.41 | 21.83 |
| X5P.c + R5P.c <=> GAP.c + S7P.c (exch) | (>1e4, >1e4) | 0 | >10000 |
| S7P.c + GAP.c <=> E4P.c + F6P.c (net) | 14.533 | 7.41 | 21.83 |
| S7P.c + GAP.c <=> E4P.c + F6P.c (exch) | 5.97 | 0 | 24.83 |
| X5P.c + E4P.c <=> GAP.c + F6P (net) | 14.533 | 7.41 | 21.83 |
| X5P.c + E4P.c <=> GAP.c + F6P (exch) | 0 | 0 | 23.39 |
| Pyr.c <=> Lact.c (net) | 319.933 | 231.31 | 407.98 |
| Pyr.c <=> Lact.c (exch) | (>1000, >1e4) | 233.28 | >10000 |
| Pyr.c -> CO2 + AcCoA.m | 65.162 | 51.32 | 81.11 |
| AcCoA.m + OAC.m -> Cit.m | 65.162 | 51.32 | 81.11 |
| Cit.m <=> AKG.m + CO2 (net) | 45.885 | 32.23 | 61.56 |
| Cit.m <=> AKG.m + CO2 (exch) | 4.757 | 1.55 | 8.22 |
| AKG.m -> CO2 + Suc.m | 72.238 | 48.32 | 99.57 |
| Suc.m <=> Fum.m (net) | 72.238 | 48.32 | 99.57 |
| Suc.m <=> Fum.m (exch) | (>1e4, >1e4) | >10000 | >10000 |
| Fum.m <=> Mal.m (net) | 72.238 | 48.32 | 99.57 |
| Fum.m <=> Mal.m (exch) | (198.0, 257.8) | 101.87 | >10000 |
| Mal.m <=> OAA.m (net) | 14.824 | 7.06 | 21.41 |
| Mal.m <=> OAA.m (exch) | 386.058 | 171.19 | >1000 |
| Gln.c -> Glu.c | 40.749 | 29.66 | 53.29 |

| Reaction | Flux | 95% Confidence Interval | |
|---|---|---|---|
| | nmol/$10^6$ cells/h | Lower Bound | Upper Bound |
| Ser.c <=> Pyr.c (net) | 16.406 | 8.58 | 24.27 |
| Ser.c <=> Pyr.c (exch) | (>1000, >1e4) | 0 | >10000 |
| Gln.ext -> Gln.c | 43.438 | 32.29 | 55.94 |
| Asp.c -> Asp.ext | 6.485 | 3.95 | 9.02 |
| Ser.ext -> Ser.c | 19.991 | 12.2 | 27.8 |
| Ala.c -> Ala.ext | 14.004 | 9.52 | 18.49 |
| Glu.c -> Glu.ext | 11.035 | 7.15 | 14.92 |
| Lact.c -> Lact.ext | 319.933 | 231.31 | 407.98 |
| Glu.c <=> AKG.m (net) | -2.426 | -14.09 | 10.59 |
| Glu.c <=> AKG.m (exch) | (0.0, 865.7) | 0 | >1000 |
| Mal.m -> Pyr.c + CO2 | 57.414 | 32.1 | 87.68 |
| Cit.m -> OAA.m + AcCoA.c | 19.277 | 15.59 | 22.97 |
| Pyr.c + CO2 -> OAA.m | 40.683 | 25.73 | 59.39 |
| 0.95 AcCoA.c + 0.05 DHAP.c -> Lipid | 20.291 | 16.41 | 24.18 |
| Pyr.c + Glu.c <=> Ala.c + AKG.m (net) | 19.157 | 14.56 | 23.76 |
| Pyr.c + Glu.c <=> Ala.c + AKG.m (exch) | (0.0, 865.7) | 0 | >10000 |
| OAA.m + Glu.c <=> Asp.c + AKG.m (net) | 9.622 | 7.01 | 12.23 |
| OAA.m + Glu.c <=> Asp.c + AKG.m (exch) | (0.0, 865.7) | 0 | >10000 |
| 0.23 Ala.c + 0.14 Asp.c + 0.12 Gln.c + 0.15 Glu.c + 0.16 Ser.c + 0.11 G6P.c + 0.09 R5P.c -> Biomass | 22.408 | 17.93 | 26.89 |
| Lact.c + Lact.tr -> Lact.c + Lact.snk | 32.816 | 7.77 | 63.01 |
| Glu.c <=> AKG.m (total) | 26.353 | | |
| Net CO2 production | 245.632 | 179.25 | 321.85 |

**Table K.** Metabolic flux analysis results for MCF7 high-lactate cultures using [1,2-$^{13}$C] glucose and [U-$^{13}$C] glutamine. The fluxes are shown with 95% confidence intervals. The sum of squared residuals (SSR) was 67.7 with an expected range of 30.8 to 69.0.

| **Reaction** | **Flux** | **95% Confidence Interval** | |
|---|---|---|---|
| | nmol/10$^6$ cells/h | Lower Bound | Upper Bound |
| Gluc.ext -> G6P.c | 157.959 | 96.22 | 170.61 |
| G6P.c <=> F6P.c (net) | 118.7 | 70.41 | 132.1 |
| G6P.c <=> F6P.c (exch) | (>1e4, >1e4) | 0 | >10000 |
| F6P.c -> FBP.c | 141.752 | 84.68 | 153.61 |
| FBP.c <=> DHAP.c + GAP.c (net) | 141.752 | 84.68 | 153.61 |
| FBP.c <=> DHAP.c + GAP.c (exch) | (0.0, >1e4) | 0 | >10000 |
| DHAP.c <=> GAP.c (net) | 141.081 | 84.19 | 152.92 |
| DHAP.c <=> GAP.c (exch) | 517.276 | 291.86 | 613.93 |
| GAP.c <=> 3PG.c (net) | 294.359 | 175.81 | 318.7 |
| GAP.c <=> 3PG.c (exch) | (0.0, >1e4) | 0 | >10000 |
| 3PG.c -> Pyr.c | 294.359 | 175.81 | 318.7 |
| G6P.c -> CO2 + Ru5P.c | 36.684 | 13.11 | 43.82 |
| Ru5P.c <=> R5P.c (net) | 13.632 | 5.75 | 16.03 |
| Ru5P.c <=> R5P.c (exch) | (0.0, >1e4) | 0 | >10000 |
| Ru5P.c <=> X5P.c (net) | 23.052 | 7.34 | 27.81 |
| Ru5P.c <=> X5P.c (exch) | (0.0,  45.4) | 0 | >10000 |
| X5P.c + R5P.c <=> GAP.c + S7P.c (net) | 11.526 | 3.67 | 13.9 |
| X5P.c + R5P.c <=> GAP.c + S7P.c (exch) | (415.8, 415.8) | 0 | >10000 |
| S7P.c + GAP.c <=> E4P.c + F6P.c (net) | 11.526 | 3.67 | 13.9 |
| S7P.c + GAP.c <=> E4P.c + F6P.c (exch) | (0.0, 0.4) | 0 | >10000 |
| X5P.c + E4P.c <=> GAP.c + F6P (net) | 11.526 | 3.67 | 13.9 |
| X5P.c + E4P.c <=> GAP.c + F6P (exch) | 0 | 0 | 13.76 |
| Pyr.c <=> Lact.c (net) | 253.443 | 139.96 | 277.5 |
| Pyr.c <=> Lact.c (exch) | 405.053 | 61.34 | 907.77 |
| Pyr.c -> CO2 + AcCoA.m | 48.238 | 36.79 | 53.83 |
| AcCoA.m + OAC.m -> Cit.m | 48.238 | 36.79 | 53.83 |
| Cit.m <=> AKG.m + CO2 (net) | 35.486 | 26.93 | 40.46 |
| Cit.m <=> AKG.m + CO2 (exch) | 19.249 | 14.16 | 21.84 |
| AKG.m -> CO2 + Suc.m | 54.276 | 39.6 | 62.37 |
| Suc.m <=> Fum.m (net) | 54.276 | 39.6 | 62.37 |
| Suc.m <=> Fum.m (exch) | (155.9, 221.5) | 97.48 | >10000 |
| Fum.m <=> Mal.m (net) | 54.276 | 39.6 | 62.37 |
| Fum.m <=> Mal.m (exch) | (>1e4, >1e4) | >10000 | >10000 |
| Mal.m <=> OAA.m (net) | 23.197 | 18.3 | 26.37 |
| Mal.m <=> OAA.m (exch) | (>1e4, >1e4) | 438.92 | >10000 |
| Gln.c -> Glu.c | 36.246 | 28.23 | 40.55 |
| Ser.c <=> Pyr.c (net) | 16.022 | 7.21 | 27.67 |
| Ser.c <=> Pyr.c (exch) | (197.4, >1e4) | 0 | >10000 |
| Gln.ext -> Gln.c | 39.055 | 30.93 | 43.37 |
| Asp.c -> Asp.ext | 6.199 | 4.06 | 8.3 |

| **Reaction** | **Flux** | **95% Confidence Interval** | |
|---|---|---|---|
| | nmol/$10^6$ cells/h | Lower Bound | Upper Bound |
| Ser.ext -> Ser.c | 19.766 | 10.95 | 31.38 |
| Ala.c -> Ala.ext | 12.632 | 7.88 | 16.85 |
| Glu.c -> Glu.ext | 13.946 | 9.41 | 18.26 |
| Lact.c -> Lact.ext | 253.443 | 139.96 | 277.5 |
| Glu.c <=> AKG.m (net) | -8.701 | -17.65 | -3.55 |
| Glu.c <=> AKG.m (exch) | (0.0, 105.3) | 0 | 126.99 |
| Mal.m -> Pyr.c + CO2 | 31.078 | 18.26 | 38.47 |
| Cit.m -> OAA.m + AcCoA.c | 12.751 | 8.67 | 15.64 |
| Pyr.c + CO2 -> OAA.m | 21.764 | 15.58 | 25.74 |
| 0.95 AcCoA.c + 0.05 DHAP.c -> Lipid | 13.423 | 9.13 | 16.46 |
| Pyr.c + Glu.c <=> Ala.c + AKG.m (net) | 18.015 | 13.1 | 22.29 |
| Pyr.c + Glu.c <=> Ala.c + AKG.m (exch) | 92.939 | 0 | 126.99 |
| OAA.m + Glu.c <=> Asp.c + AKG.m (net) | 9.475 | 7.22 | 11.62 |
| OAA.m + Glu.c <=> Asp.c + AKG.m (exch) | (0.0, 105.3) | 0 | 126.99 |
| 0.23 Ala.c + 0.14 Asp.c + 0.12 Gln.c + 0.15 Glu.c + 0.16 Ser.c + 0.11 G6P.c + 0.09 R5P.c -> Biomass | 23.404 | 18.9 | 27.46 |
| Lact.c + Lact.tr -> Lact.c + Lact.snk | 65.652 | 20.76 | 83.1 |
| Glu.c <=> AKG.m (total) | 18.789 | | |
| Net CO2 production | 183.998 | 130.52 | 205.72 |

**Table L.** Metabolic flux analysis results for MDA-MB-231 control cultures using [1,2-$^{13}$C] glucose and [U-$^{13}$C] glutamine. The fluxes are shown with 95% confidence intervals. The sum of squared residuals (SSR) was 62.1 with an expected range of 34.8 to 75.0.

| **Reaction** | **Flux** | **95% Confidence Interval** | |
|---|---|---|---|
| | nmol/$10^6$ cells/h | Lower Bound | Upper Bound |
| Gluc.ext -> G6P.c | 323.662 | 241.31 | 407.08 |
| G6P.c <=> F6P.c (net) | 238.695 | 177.95 | 313.69 |
| G6P.c <=> F6P.c (exch) | (>1e4, >1e4) | 0 | >10000 |
| F6P.c -> FBP.c | 291.889 | 217.43 | 368.7 |
| FBP.c <=> DHAP.c + GAP.c (net) | 291.889 | 217.43 | 368.7 |
| FBP.c <=> DHAP.c + GAP.c (exch) | (0.0, >1e4) | 0 | >10000 |
| DHAP.c <=> GAP.c (net) | 290.671 | 216.23 | 367.46 |
| DHAP.c <=> GAP.c (exch) | 1005.797 | 691.44 | >1000 |
| GAP.c <=> 3PG.c (net) | 609.157 | 452.44 | 768.83 |
| GAP.c <=> 3PG.c (exch) | (0.0, >1e4) | 0 | >10000 |
| 3PG.c -> Pyr.c | 609.157 | 452.44 | 768.83 |
| G6P.c -> CO2 + Ru5P.c | 82.12 | 42.77 | 114.93 |
| Ru5P.c <=> R5P.c (net) | 28.926 | 15.8 | 39.87 |
| Ru5P.c <=> R5P.c (exch) | (0.0, >1e4) | 0 | >10000 |
| Ru5P.c <=> X5P.c (net) | 53.193 | 26.96 | 75.06 |
| Ru5P.c <=> X5P.c (exch) | (0.0, >1e4) | 0 | >10000 |
| X5P.c + R5P.c <=> GAP.c + S7P.c (net) | 26.597 | 13.48 | 37.53 |
| X5P.c + R5P.c <=> GAP.c + S7P.c (exch) | (>1e4, >1e4) | 0 | >10000 |
| S7P.c + GAP.c <=> E4P.c + F6P.c (net) | 26.597 | 13.48 | 37.53 |
| S7P.c + GAP.c <=> E4P.c + F6P.c (exch) | 2.36 | 0 | 31.22 |
| X5P.c + E4P.c <=> GAP.c + F6P (net) | 26.597 | 13.48 | 37.53 |
| X5P.c + E4P.c <=> GAP.c + F6P (exch) | 0 | 0 | 39.96 |
| Pyr.c <=> Lact.c (net) | 563.768 | 407.51 | 723.24 |
| Pyr.c <=> Lact.c (exch) | (0.0, >1e4) | 0 | >10000 |
| Pyr.c -> CO2 + AcCoA.m | 51.947 | 40.32 | 66.36 |
| AcCoA.m + OAC.m -> Cit.m | 51.947 | 40.32 | 66.36 |
| Cit.m <=> AKG.m + CO2 (net) | 28.816 | 18.37 | 43.1 |
| Cit.m <=> AKG.m + CO2 (exch) | 6.307 | 4.27 | 8.98 |
| AKG.m -> CO2 + Suc.m | 46.494 | 26.68 | 73.21 |
| Suc.m <=> Fum.m (net) | 46.494 | 26.68 | 73.21 |
| Suc.m <=> Fum.m (exch) | 92.427 | 43.99 | 212.9 |
| Fum.m <=> Mal.m (net) | 46.494 | 26.68 | 73.21 |
| Fum.m <=> Mal.m (exch) | (>1000, >1e4) | 266.76 | >10000 |
| Mal.m <=> OAA.m (net) | 12.57 | 9.22 | 16 |
| Mal.m <=> OAA.m (exch) | 353.445 | 161.88 | >1000 |
| Gln.c -> Glu.c | 30.163 | 20.23 | 43 |
| Ser.c <=> Pyr.c (net) | 11.898 | 5.22 | 18.51 |
| Ser.c <=> Pyr.c (exch) | (>1e4, >1e4) | 0 | >10000 |
| Gln.ext -> Gln.c | 33.269 | 23.28 | 46.2 |

| Reaction | Flux | 95% Confidence Interval | |
|---|---|---|---|
| | nmol/$10^6$ cells/h | Lower Bound | Upper Bound |
| Asp.c -> Asp.ext | 3.451 | 2.09 | 4.81 |
| Ser.ext -> Ser.c | 16.039 | 9.41 | 22.62 |
| Ala.c -> Ala.ext | 9.989 | 6.48 | 13.5 |
| Glu.c -> Glu.ext | 8.603 | 5.49 | 11.72 |
| Lact.c -> Lact.ext | 563.768 | 407.51 | 723.24 |
| Glu.c <=> AKG.m (net) | -5.34 | -15.81 | 7.66 |
| Glu.c <=> AKG.m (exch) | (0.0, 628.4) | 0 | >1000 |
| Mal.m -> Pyr.c + CO2 | 33.924 | 14.38 | 60.72 |
| Cit.m -> OAA.m + AcCoA.c | 23.131 | 19.07 | 27.23 |
| Pyr.c + CO2 -> OAA.m | 23.321 | 13.27 | 37.66 |
| 0.95 AcCoA.c + 0.05 DHAP.c -> Lipid | 24.348 | 20.08 | 28.67 |
| Pyr.c + Glu.c <=> Ala.c + AKG.m (net) | 15.943 | 12.25 | 19.64 |
| Pyr.c + Glu.c <=> Ala.c + AKG.m (exch) | (0.0, 628.4) | 0 | >1000 |
| OAA.m + Glu.c <=> Asp.c + AKG.m (net) | 7.075 | 5.54 | 8.61 |
| OAA.m + Glu.c <=> Asp.c + AKG.m (exch) | (0.0, 628.4) | 0 | >1000 |
| 0.23 Ala.c + 0.14 Asp.c + 0.12 Gln.c + 0.15 Glu.c + 0.16 Ser.c + 0.11 G6P.c + 0.09 R5P.c -> Biomass | 25.884 | 20.82 | 30.95 |
| Lact.c + Lact.tr -> Lact.c + Lact.snk | 2.1 | 0 | 31.21 |
| Glu.c <=> AKG.m (total) | 17.678 | | |
| Net CO2 production | 219.979 | 152.69 | 294.91 |

**Table M.** Metabolic flux analysis results for MDA-MB-231 high-lactate cultures using [1,2- $^{13}$C] glucose and [U-$^{13}$C] glutamine. The fluxes are shown with 95% confidence intervals. The sum of squared residuals (SSR) was 38.3 with an expected range of 34.8 to 75.0.

| Reaction | Flux | 95% Confidence Interval | |
|---|---|---|---|
| | nmol/$10^6$ cells/h | Lower Bound | Upper Bound |
| Gluc.ext -> G6P.c | 269.671 | 209.66 | 333.36 |
| G6P.c <=> F6P.c (net) | 213.317 | 164.04 | 266.24 |
| G6P.c <=> F6P.c (exch) | (>1e4, >1e4) | 0 | >10000 |
| F6P.c -> FBP.c | 247.391 | 191.86 | 306.37 |
| FBP.c <=> DHAP.c + GAP.c (net) | 247.391 | 191.86 | 306.37 |
| FBP.c <=> DHAP.c + GAP.c (exch) | (0.0, >1e4) | 0 | >10000 |
| DHAP.c <=> GAP.c (net) | 246.347 | 190.85 | 305.29 |
| DHAP.c <=> GAP.c (exch) | (>1e4, >1e4) | >1000 | >10000 |
| GAP.c <=> 3PG.c (net) | 510.774 | 395.1 | 632.72 |
| GAP.c <=> 3PG.c (exch) | (0.0, >1e4) | 0 | >10000 |
| 3PG.c -> Pyr.c | 510.774 | 395.1 | 632.72 |
| G6P.c -> CO2 + Ru5P.c | 53.47 | 31.02 | 74.99 |
| Ru5P.c <=> R5P.c (net) | 19.397 | 11.96 | 26.58 |
| Ru5P.c <=> R5P.c (exch) | (0.0, >1e4) | 0 | >10000 |
| Ru5P.c <=> X5P.c (net) | 34.074 | 19.09 | 48.42 |
| Ru5P.c <=> X5P.c (exch) | (0.0, >1e4) | 0 | >10000 |
| X5P.c + R5P.c <=> GAP.c + S7P.c (net) | 17.037 | 9.54 | 24.21 |
| X5P.c + R5P.c <=> GAP.c + S7P.c (exch) | (0.0, 4.3) | 0 | >10000 |
| S7P.c + GAP.c <=> E4P.c + F6P.c (net) | 17.037 | 9.54 | 24.21 |
| S7P.c + GAP.c <=> E4P.c + F6P.c (exch) | (>1000, >1e4) | 0 | >10000 |
| X5P.c + E4P.c <=> GAP.c + F6P (net) | 17.037 | 9.54 | 24.21 |
| X5P.c + E4P.c <=> GAP.c + F6P (exch) | 0.791 | 0 | 24.62 |
| Pyr.c <=> Lact.c (net) | 459.32 | 346.09 | 578.84 |
| Pyr.c <=> Lact.c (exch) | (>1000, >1000) | >1000 | >10000 |
| Pyr.c -> CO2 + AcCoA.m | 84.266 | 68.37 | 102.99 |
| AcCoA.m + OAC.m -> Cit.m | 84.266 | 68.37 | 102.99 |
| Cit.m <=> AKG.m + CO2 (net) | 64.437 | 49.71 | 82.34 |
| Cit.m <=> AKG.m + CO2 (exch) | 31.16 | 24.15 | 39.76 |
| AKG.m -> CO2 + Suc.m | 104.519 | 79.32 | 135.31 |
| Suc.m <=> Fum.m (net) | 104.519 | 79.32 | 135.31 |
| Suc.m <=> Fum.m (exch) | (652.8, >1000) | 263.79 | >10000 |
| Fum.m <=> Mal.m (net) | 104.519 | 79.32 | 135.31 |
| Fum.m <=> Mal.m (exch) | (>1000, >1e4) | 668.42 | >10000 |
| Mal.m <=> OAA.m (net) | 25.781 | 19.91 | 32.28 |
| Mal.m <=> OAA.m (exch) | (>1e4, >1e4) | >1000 | >10000 |
| Gln.c -> Glu.c | 54.444 | 43.11 | 68.02 |
| Ser.c <=> Pyr.c (net) | 15.336 | 4.6 | 26.08 |
| Ser.c <=> Pyr.c (exch) | (169.7, >1e4) | 0 | >10000 |
| Gln.ext -> Gln.c | 57.59 | 46.16 | 71.17 |
| Asp.c -> Asp.ext | 4.643 | 2.5 | 6.78 |

| Reaction | Flux | 95% Confidence Interval | |
|---|---|---|---|
| | nmol/$10^6$ cells/h | Lower Bound | Upper Bound |
| Ser.ext -> Ser.c | 19.531 | 8.82 | 30.24 |
| Ala.c -> Ala.ext | 8.262 | 4.36 | 12.16 |
| Glu.c -> Glu.ext | 10.428 | 5.58 | 15.27 |
| Lact.c -> Lact.ext | 459.32 | 346.09 | 578.84 |
| Glu.c <=> AKG.m (net) | 17.476 | 5.55 | 31.48 |
| Glu.c <=> AKG.m (exch) | 366.926 | 0 | >1000 |
| Mal.m -> Pyr.c + CO2 | 78.738 | 55.59 | 107.5 |
| Cit.m -> OAA.m + AcCoA.c | 19.83 | 15.75 | 23.95 |
| Pyr.c + CO2 -> OAA.m | 46.969 | 34.67 | 62.74 |
| 0.95 AcCoA.c + 0.05 DHAP.c -> Lipid | 20.873 | 16.58 | 25.21 |
| Pyr.c + Glu.c <=> Ala.c + AKG.m (net) | 14.293 | 10.22 | 18.36 |
| Pyr.c + Glu.c <=> Ala.c + AKG.m (exch) | 357.26 | 0 | >1000 |
| OAA.m + Glu.c <=> Asp.c + AKG.m (net) | 8.314 | 6.06 | 10.57 |
| OAA.m + Glu.c <=> Asp.c + AKG.m (exch) | (0.0, 964.0) | 0 | >1000 |
| 0.23 Ala.c + 0.14 Asp.c + 0.12 Gln.c + 0.15 Glu.c + 0.16 Ser.c + 0.11 G6P.c + 0.09 R5P.c -> Biomass | 26.22 | 21.15 | 31.31 |
| Lact.c + Lact.tr -> Lact.c + Lact.snk | 147.176 | 96.59 | 216.76 |
| Glu.c <=> AKG.m (total) | 40.083 | | |
| Net CO2 production | 338.461 | 261.22 | 428.14 |

**Table N.** Metabolic flux analysis results for MCF7 control cultures using [1,2-$^{13}$C] glucose and [U-$^{13}$C] glutamine using the 0.6 mol% standard error for the MIDs for intracellular glutamine, where the biological replicates had high standard error. The fluxes are shown with 95% confidence intervals. The sum of squared residuals (SSR) was 56.4 with an expected range of 30.8 to 69.0. **Table S10** has the metabolic flux results for this culture when the standard error for the biological replicates was used. The fluxes are not significantly different between the two simulations.

| **Reaction** | **Flux** | **95% Confidence Interval** | |
|---|---|---|---|
| | nmol/10$^6$ cells/h | Lower Bound | Upper Bound |
| Gluc.ext -> G6P.c | 197.061 | 149.98 | 243.88 |
| G6P.c <=> F6P.c (net) | 150.994 | 138.25 | 191.77 |
| G6P.c <=> F6P.c (exch) | (>1000, >1e4) | 0 | >10000 |
| F6P.c -> FBP.c | 178.712 | 135.05 | 222.12 |
| FBP.c <=> DHAP.c + GAP.c (net) | 178.712 | 135.05 | 222.12 |
| FBP.c <=> DHAP.c + GAP.c (exch) | (0.0, >1e4) | 0 | >10000 |
| DHAP.c <=> GAP.c (net) | 177.697 | 134.04 | 221.1 |
| DHAP.c <=> GAP.c (exch) | (>1000, >1000) | >1000 | >10000 |
| GAP.c <=> 3PG.c (net) | 370.268 | 279.69 | 460.17 |
| GAP.c <=> 3PG.c (exch) | (0.0, >1e4) | 0 | >10000 |
| 3PG.c -> Pyr.c | 370.268 | 279.69 | 460.17 |
| G6P.c -> CO2 + Ru5P.c | 43.598 | 22.77 | 46.94 |
| Ru5P.c <=> R5P.c (net) | 15.88 | 8.94 | 17 |
| Ru5P.c <=> R5P.c (exch) | (>1e4, >1e4) | 0 | >10000 |
| Ru5P.c <=> X5P.c (net) | 27.718 | 13.83 | 29.95 |
| Ru5P.c <=> X5P.c (exch) | (0.0, 624.4) | 0 | >10000 |
| X5P.c + R5P.c <=> GAP.c + S7P.c (net) | 13.859 | 6.91 | 14.97 |
| X5P.c + R5P.c <=> GAP.c + S7P.c (exch) | 5.122 | 0 | 24.56 |
| S7P.c + GAP.c <=> E4P.c + F6P.c (net) | 13.859 | 6.91 | 14.97 |
| S7P.c + GAP.c <=> E4P.c + F6P.c (exch) | (>1000, >1e4) | 0 | >10000 |
| X5P.c + E4P.c <=> GAP.c + F6P (net) | 13.859 | 6.91 | 14.97 |
| X5P.c + E4P.c <=> GAP.c + F6P (exch) | 0 | 0 | 61.78 |
| Pyr.c <=> Lact.c (net) | 319.073 | 229.84 | 407.69 |
| Pyr.c <=> Lact.c (exch) | (>1000, >1e4) | 200.13 | >10000 |
| Pyr.c -> CO2 + AcCoA.m | 65.08 | 51.25 | 81 |
| AcCoA.m + OAC.m -> Cit.m | 65.08 | 51.25 | 81 |
| Cit.m <=> AKG.m + CO2 (net) | 45.806 | 32.18 | 61.45 |
| Cit.m <=> AKG.m + CO2 (exch) | 4.737 | 1.54 | 8.2 |
| AKG.m -> CO2 + Suc.m | 72.082 | 48.15 | 99.54 |
| Suc.m <=> Fum.m (net) | 72.082 | 48.15 | 99.54 |
| Suc.m <=> Fum.m (exch) | (>1e4, >1e4) | >10000 | >10000 |
| Fum.m <=> Mal.m (net) | 72.082 | 48.15 | 99.54 |
| Fum.m <=> Mal.m (exch) | 223.139 | 101.45 | 923.97 |
| Mal.m <=> OAA.m (net) | 14.806 | 7.05 | 21.37 |
| Mal.m <=> OAA.m (exch) | 384.54 | 170.71 | >1000 |
| Gln.c -> Glu.c | 40.677 | 29.56 | 53.19 |

| Reaction | Flux | 95% Confidence Interval | |
|---|---|---|---|
| | nmol/$10^6$ cells/h | Lower Bound | Upper Bound |
| Ser.c <=> Pyr.c (net) | 16.404 | 8.58 | 24.22 |
| Ser.c <=> Pyr.c (exch) | (>1000, >1e4) | 0 | >10000 |
| Gln.ext -> Gln.c | 43.371 | 32.19 | 55.94 |
| Asp.c -> Asp.ext | 6.485 | 3.95 | 9.02 |
| Ser.ext -> Ser.c | 19.996 | 12.21 | 27.79 |
| Ala.c -> Ala.ext | 14.002 | 9.52 | 18.48 |
| Glu.c -> Glu.ext | 11.034 | 7.14 | 14.92 |
| Lact.c -> Lact.ext | 319.073 | 229.84 | 407.69 |
| Glu.c <=> AKG.m (net) | -2.518 | -14.21 | 10.47 |
| Glu.c <=> AKG.m (exch) | (0.0, 865.2) | 0 | >1000 |
| Mal.m -> Pyr.c + CO2 | 57.276 | 32.04 | 87.48 |
| Cit.m -> OAA.m + AcCoA.c | 19.274 | 15.57 | 22.97 |
| Pyr.c + CO2 -> OAA.m | 40.628 | 25.72 | 59.31 |
| 0.95 AcCoA.c + 0.05 DHAP.c -> Lipid | 20.289 | 16.39 | 24.17 |
| Pyr.c + Glu.c <=> Ala.c + AKG.m (net) | 19.166 | 14.55 | 23.76 |
| Pyr.c + Glu.c <=> Ala.c + AKG.m (exch) | (0.0, 865.2) | 0 | >10000 |
| OAA.m + Glu.c <=> Asp.c + AKG.m (net) | 9.628 | 7.01 | 12.24 |
| OAA.m + Glu.c <=> Asp.c + AKG.m (exch) | (0.0, 865.2) | 0 | >10000 |
| 0.23 Ala.c + 0.14 Asp.c + 0.12 Gln.c + 0.15 Glu.c + 0.16 Ser.c + 0.11 G6P.c + 0.09 R5P.c -> Biomass | 22.451 | 17.97 | 26.93 |
| Lact.c + Lact.tr -> Lact.c + Lact.snk | 31.054 | 5.9 | 64.89 |
| Glu.c <=> AKG.m (total) | 26.276 | | |
| Net CO2 production | 243.213 | 176.69 | 319.8 |

**Fig G.** Measured and simulated MIDs for intracellular metabolites from [1,2-$^{13}$C] glucose labeling for MCF 10A control cultures. MIDs shown below have been corrected for natural abundance.

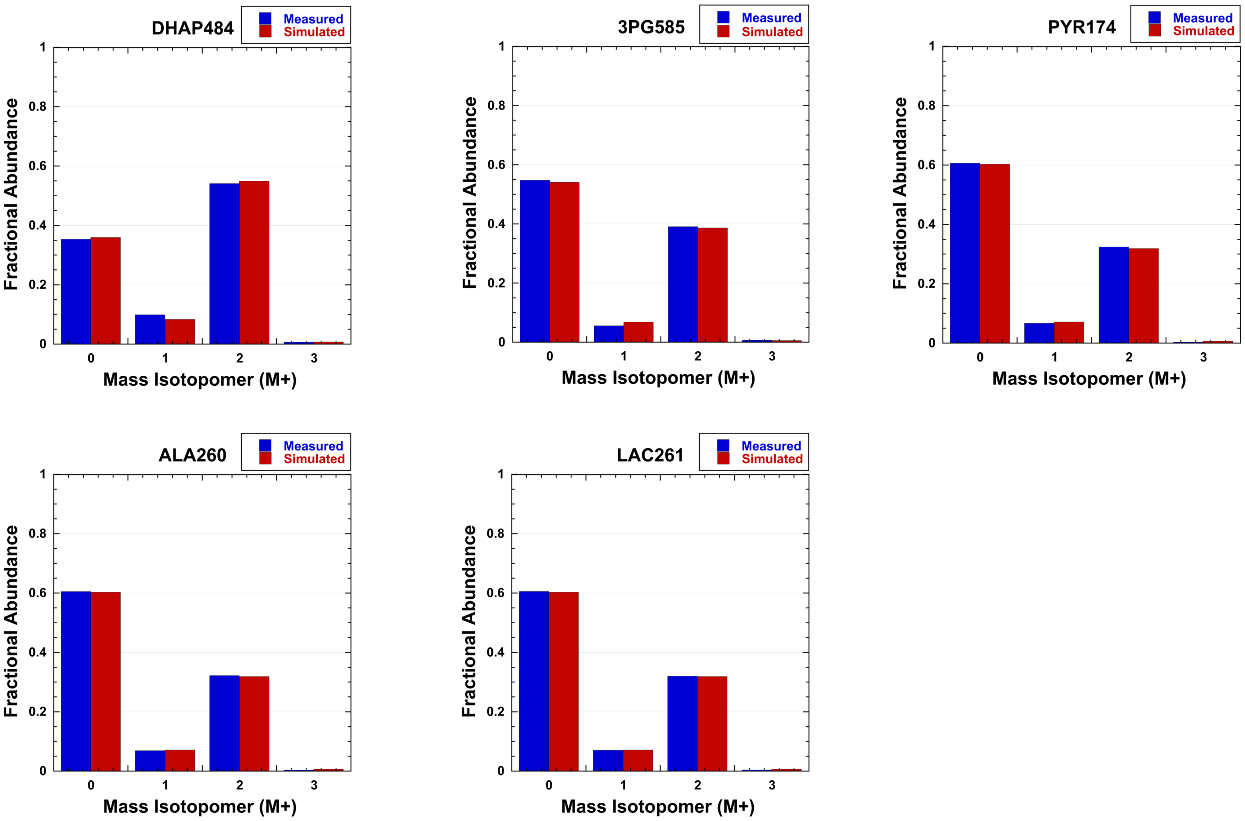

**Fig H.** Measured and simulated MIDs for intracellular metabolites from [1,2-$^{13}$C] glucose labeling for MCF 10A high-lactate cultures. MIDs shown below have been corrected for natural abundance.

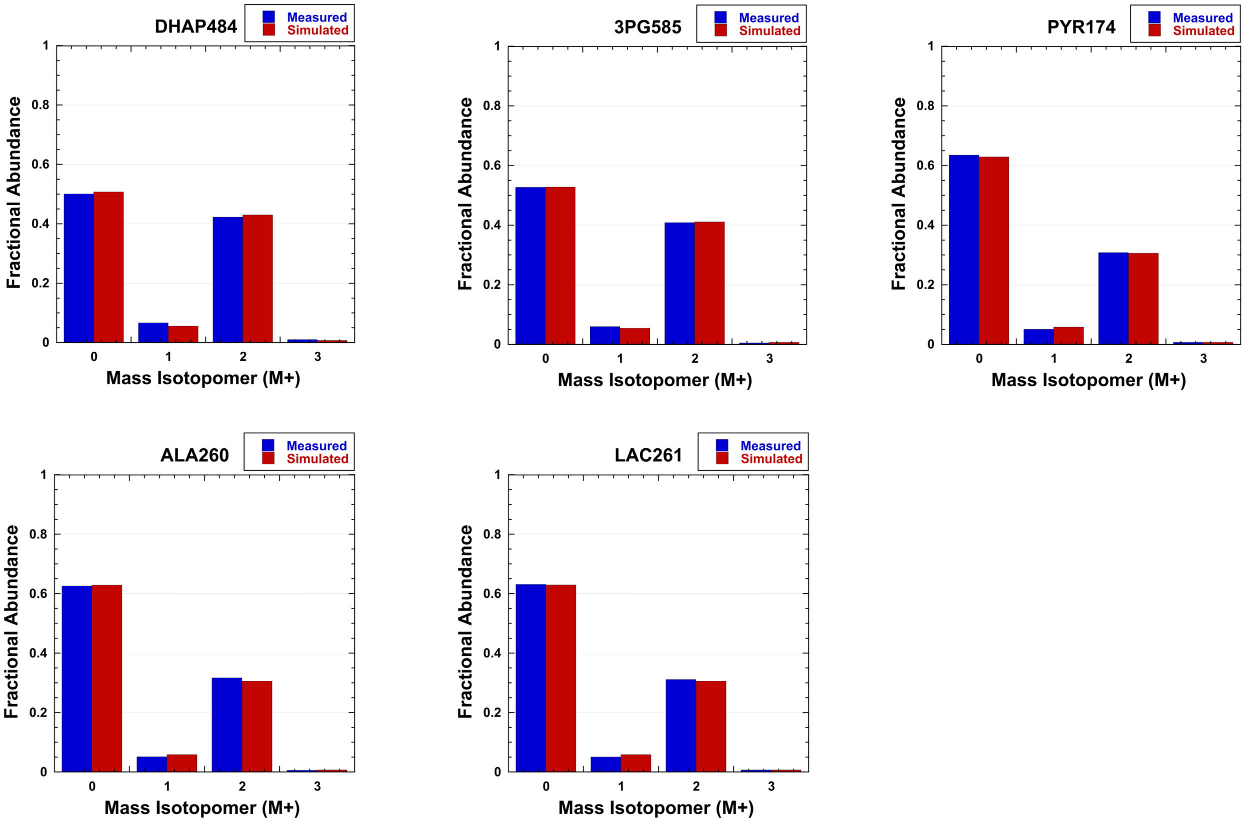

**Fig I.** Measured and simulated MIDs for intracellular metabolites from [1,2-$^{13}$C] glucose labeling for MCF7 control cultures. MIDs shown below have been corrected for natural abundance.

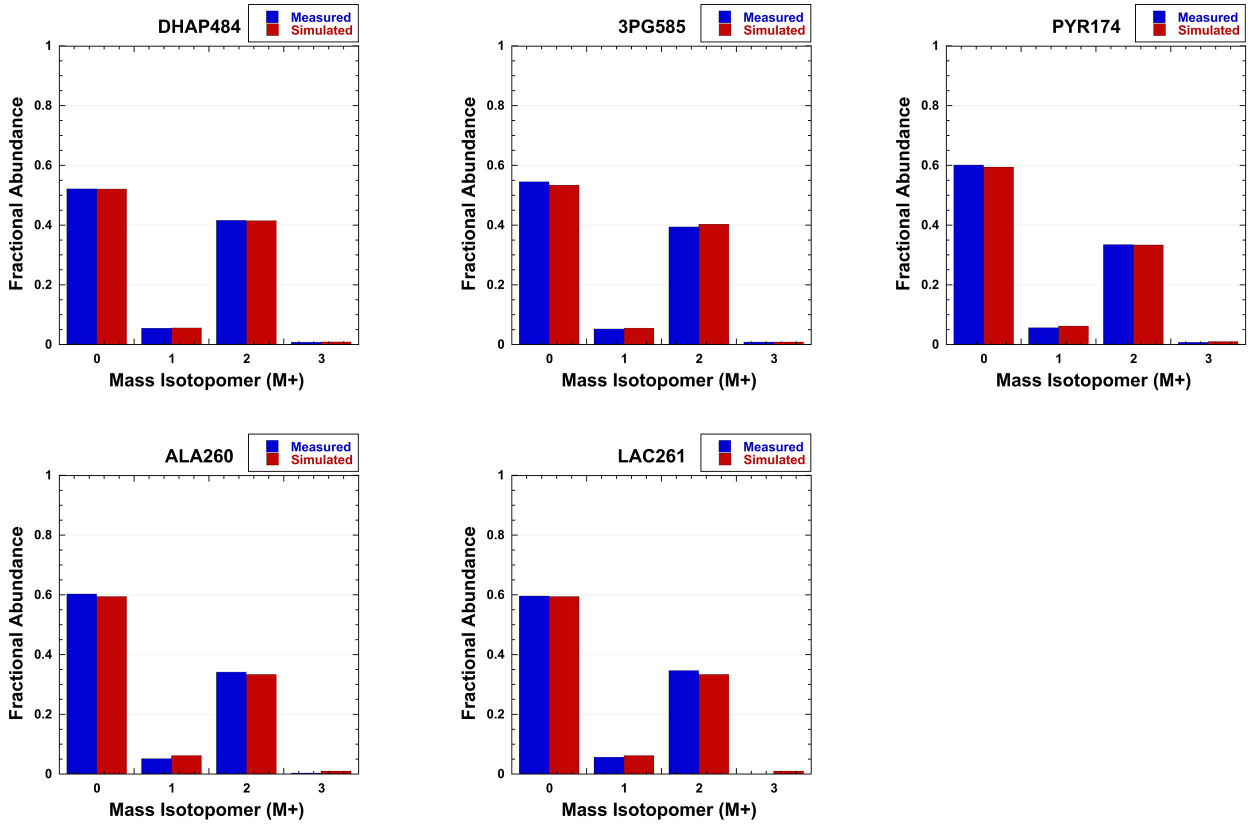

**Fig J.** Measured and simulated MIDs for intracellular metabolites from [1,2-$^{13}$C] glucose labeling for MCF7 high-lactate cultures. MIDs shown below have been corrected for natural abundance.

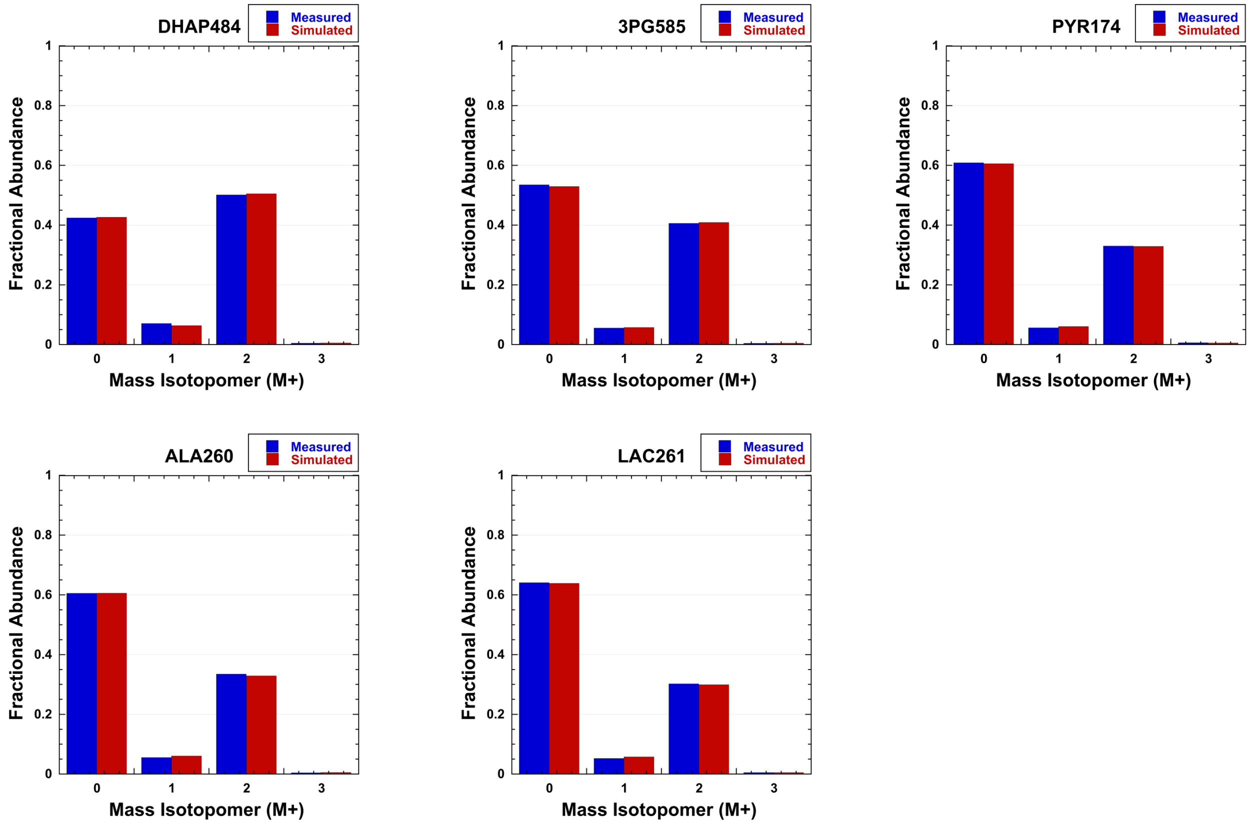

**Fig K.** Measured and simulated MIDs for intracellular metabolites from [1,2-$^{13}$C] glucose labeling for MDA-MB-231 control cultures. MIDs shown below have been corrected for natural abundance.

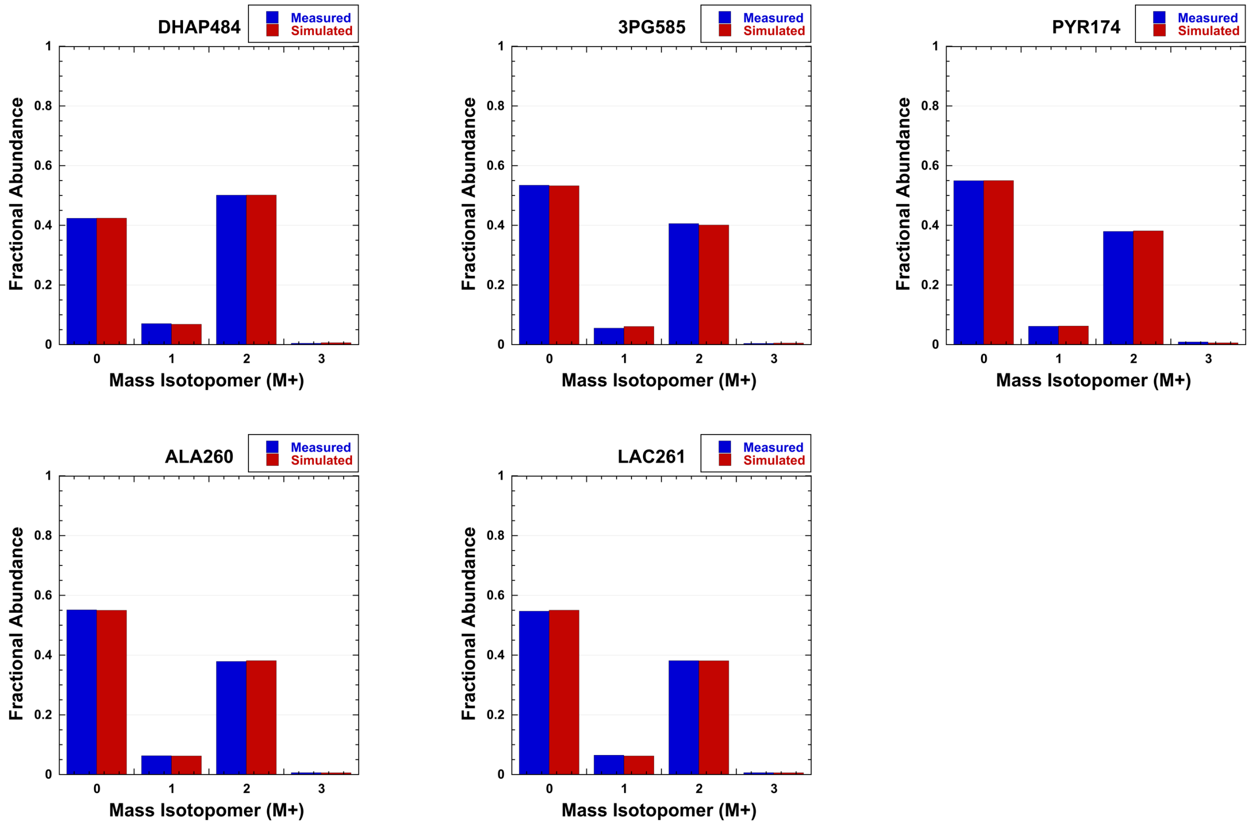

**Fig L.** Measured and simulated MIDs for intracellular metabolites from [1,2-$^{13}$C] glucose labeling for MDA-MB-231 high-lactate cultures. MIDs shown below have been corrected for natural abundance.

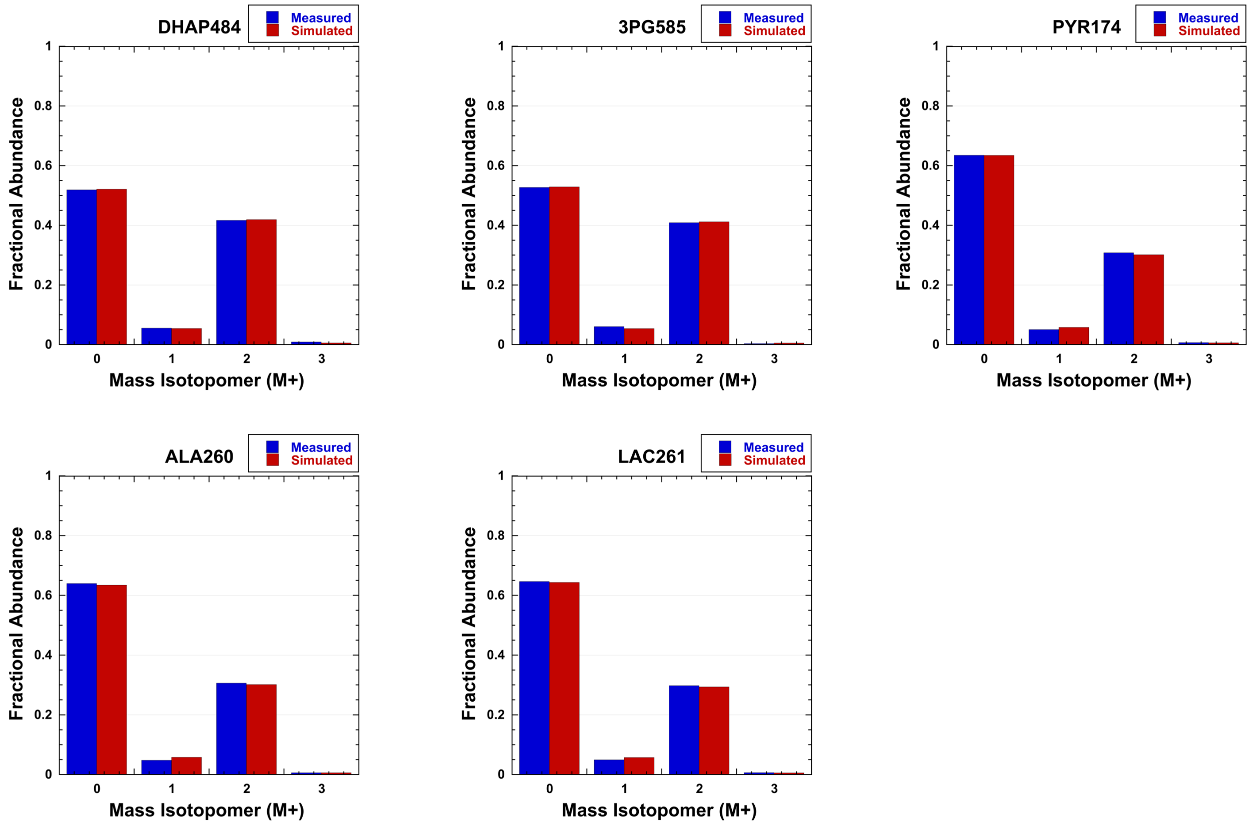

**Fig M.** Measured and simulated MIDs for intracellular metabolites from [U-$^{13}$C] glutamine labeling for MCF 10A control cultures. MIDs shown below have been corrected for natural abundance.

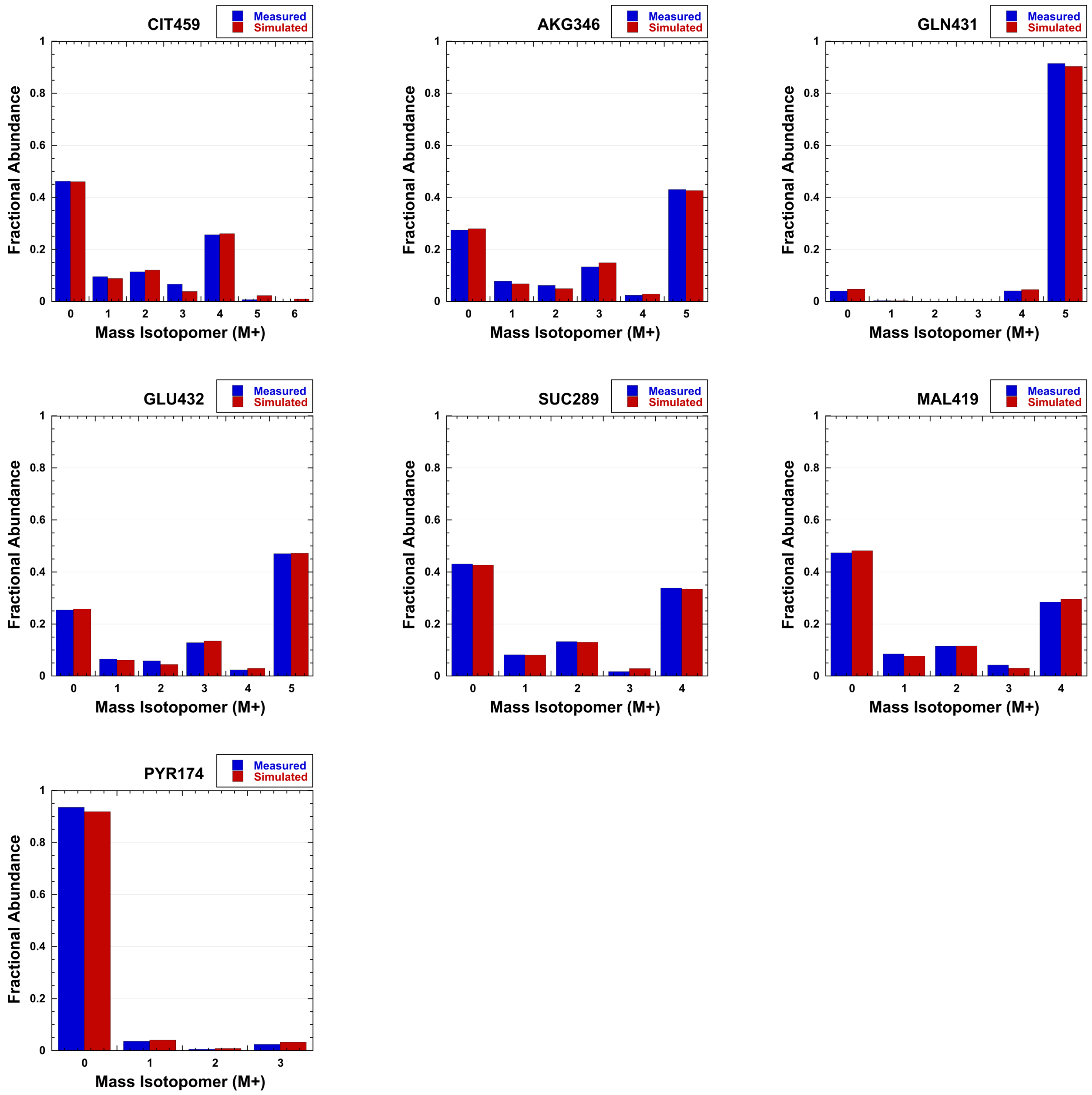

**Fig N.** Measured and simulated MIDs for intracellular metabolites from [U-$^{13}$C] glutamine labeling for MCF 10A high-lactate cultures. MIDs shown below have been corrected for natural abundance.

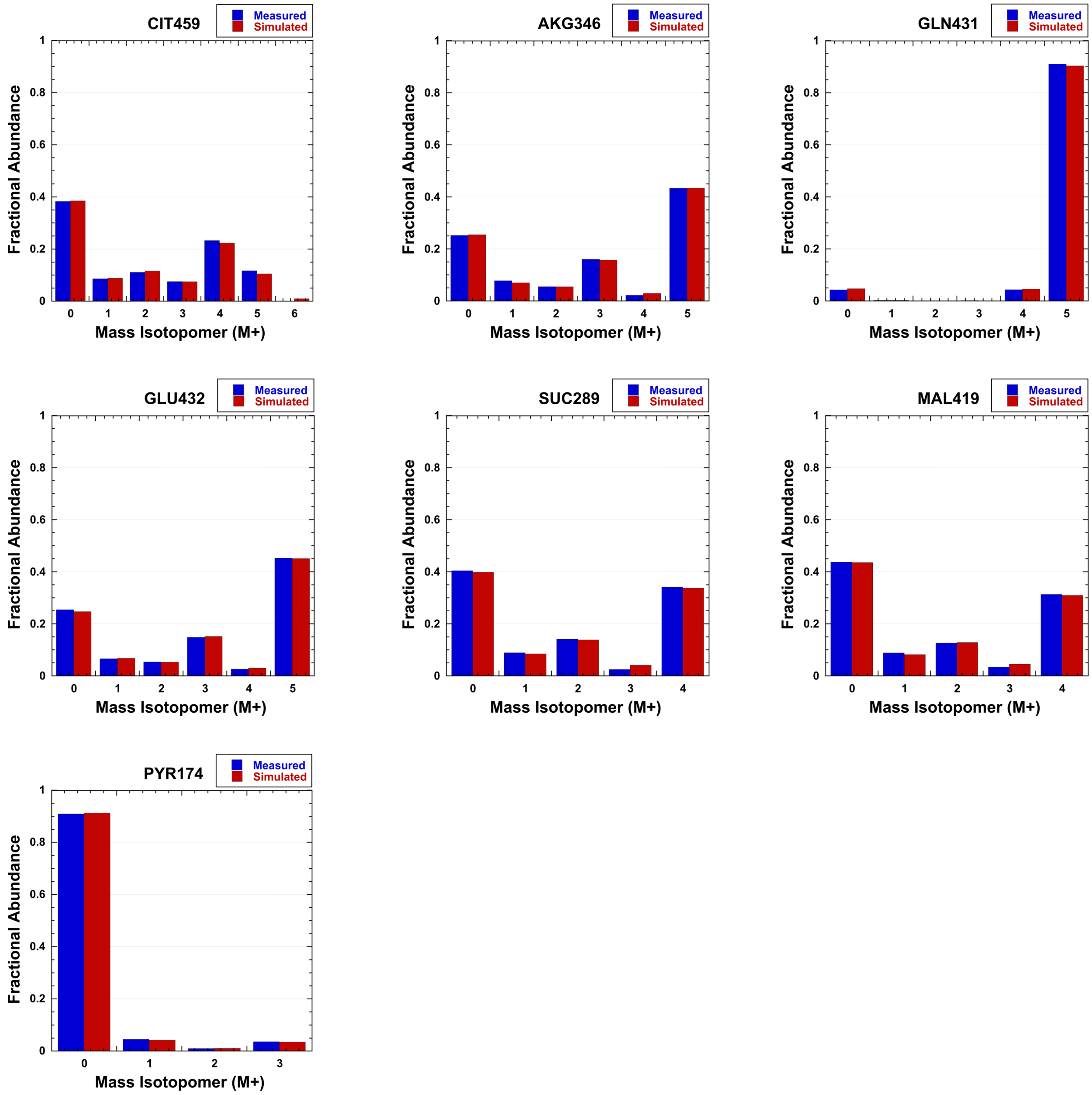

**Fig O.** Measured and simulated MIDs for intracellular metabolites from [U-$^{13}$C] glutamine labeling for MCF7 control cultures. MIDs shown below have been corrected for natural abundance.

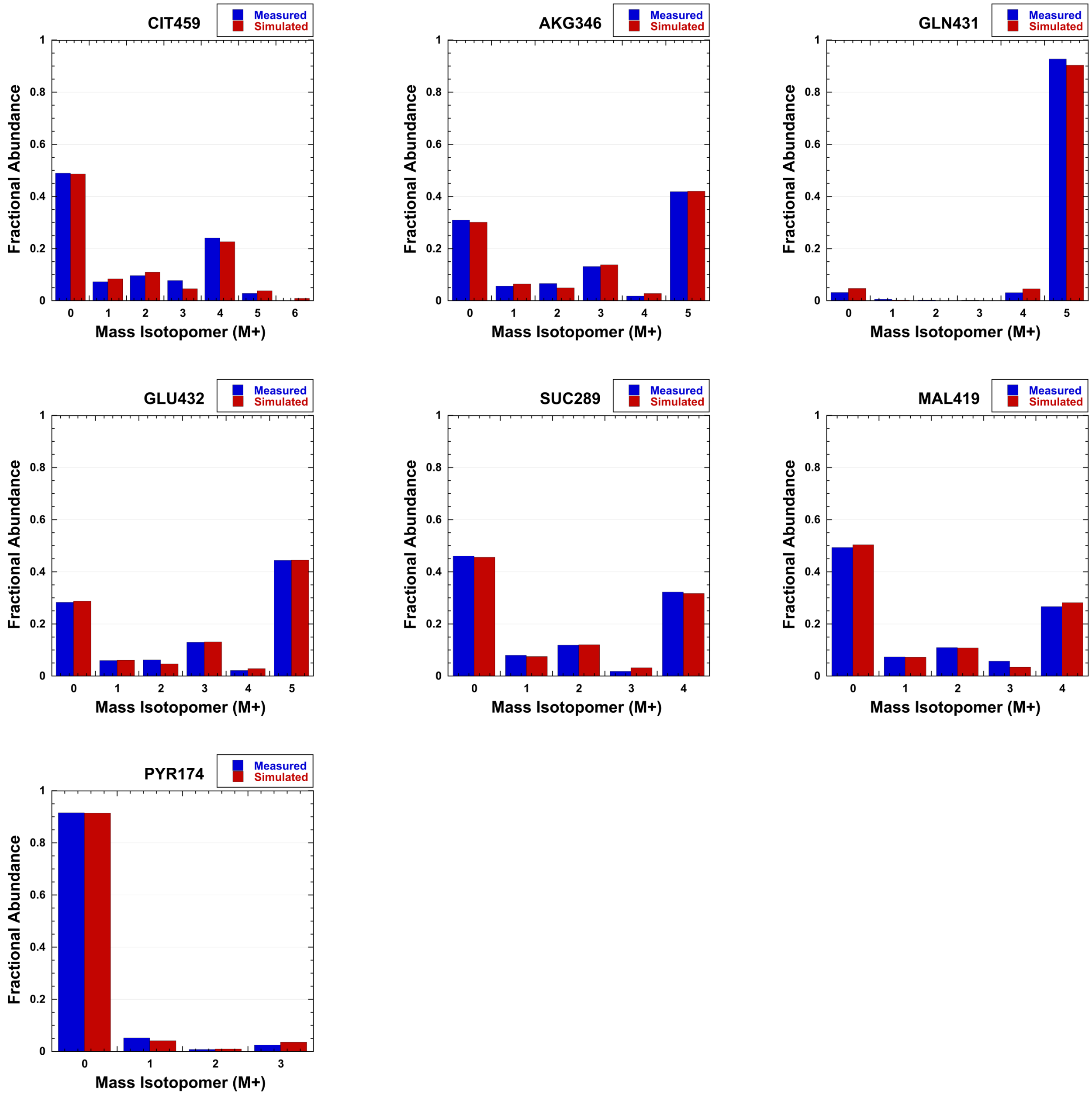

**Fig P.** Measured and simulated MIDs for intracellular metabolites from [U-$^{13}$C] glutamine labeling for MCF7 high-lactate cultures. MIDs shown below have been corrected for natural abundance.

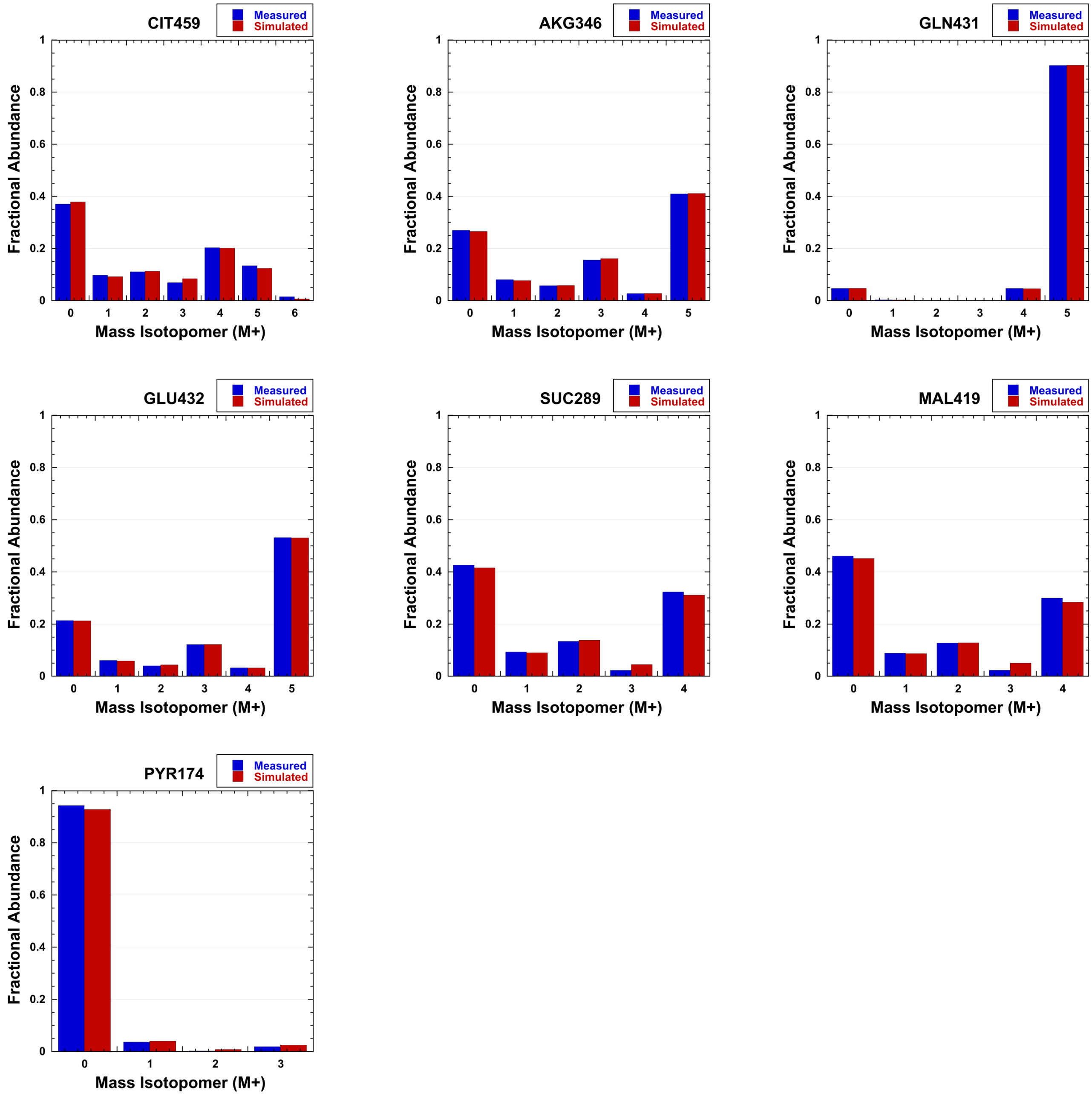

**Fig Q.** Measured and simulated MIDs for intracellular metabolites from [U-$^{13}$C] glutamine labeling for MDA-MB-231 control cultures. MIDs shown below have been corrected for natural abundance.

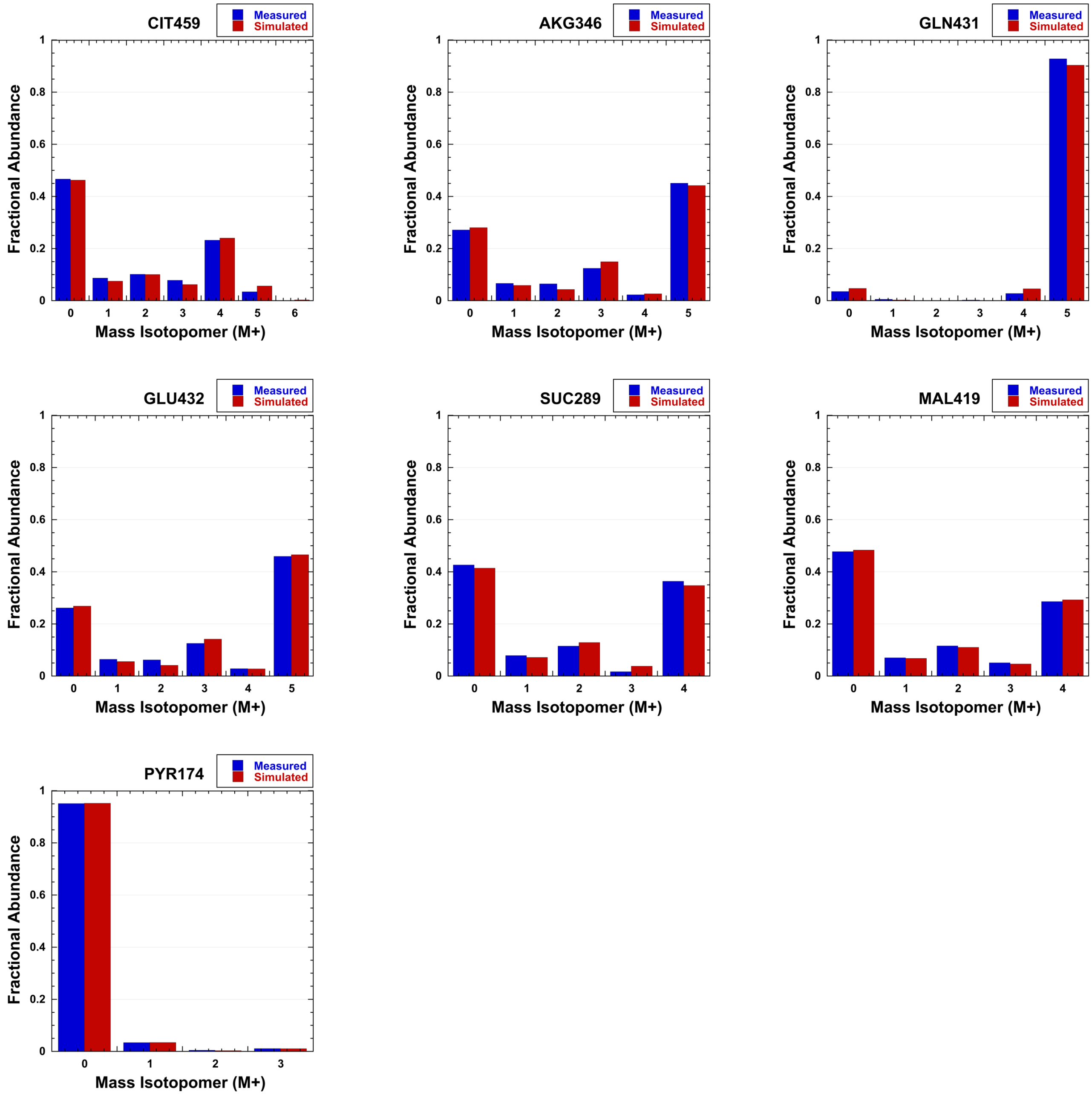

**Fig R.** Measured and simulated MIDs for intracellular metabolites from [U-$^{13}$C] glutamine labeling for MDA-MB-231 high-lactate cultures. MIDs shown below have been corrected for natural abundance.

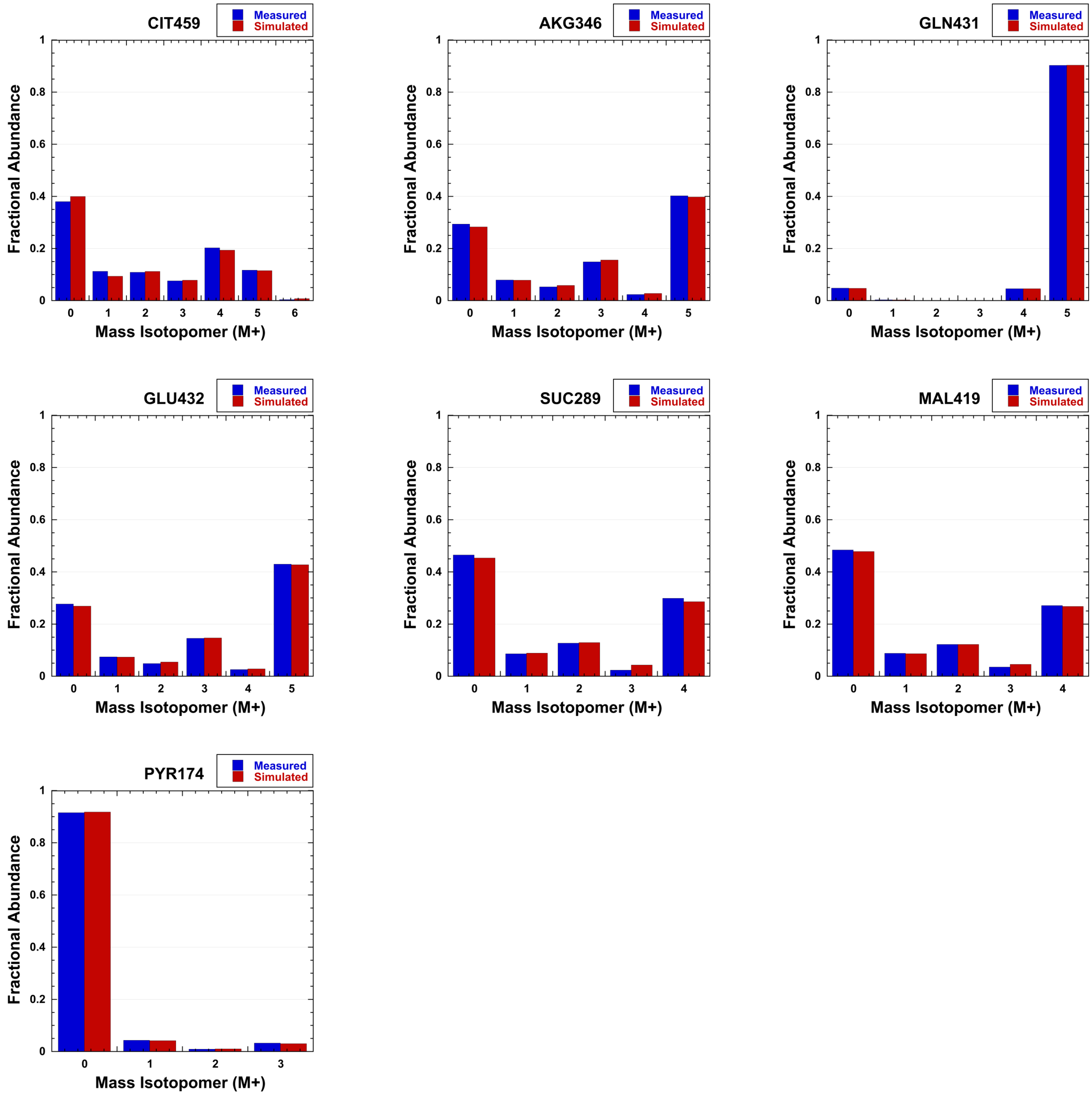

**Supplement References**

1. Ahn WS, Antoniewicz MRJMe. Parallel labeling experiments with [1, 2-$^{13}$C] glucose and [U-$^{13}$C] glutamine provide new insights into CHO cell metabolism. 2013;15:34-47.
2. Metallo CM, Walther JL, Stephanopoulos G. Evaluation of $^{13}$C isotopic tracers for metabolic flux analysis in mammalian cells. J Biotechnol. 2009;144(3):167-74. doi: 10.1016/j.jbiotec.2009.07.010. PubMed PMID: WOS:000272861600002.
